# Plant Omics Data Center: An Integrated Web Repository for Interspecies Gene Expression Networks with NLP-Based Curation

**DOI:** 10.1093/pcp/pcu188

**Published:** 2014-12-11

**Authors:** Hajime Ohyanagi, Tomoyuki Takano, Shin Terashima, Masaaki Kobayashi, Maasa Kanno, Kyoko Morimoto, Hiromi Kanegae, Yohei Sasaki, Misa Saito, Satomi Asano, Soichi Ozaki, Toru Kudo, Koji Yokoyama, Koichiro Aya, Keita Suwabe, Go Suzuki, Koh Aoki, Yasutaka Kubo, Masao Watanabe, Makoto Matsuoka, Kentaro Yano

**Affiliations:** ^1^School of Agriculture, Meiji University, Kawasaki, 214-8571 Japan; ^2^CREST, JST, Saitama, 332-0012 Japan; ^3^Tsukuba Division, Mitsubishi Space Software Co., Ltd., Tsukuba, 305-0032 Japan; ^4^Plant Genetics Laboratory, National Institute of Genetics, Mishima, 411-8540 Japan; ^5^Bioscience and Biotechnology Center, Nagoya University, Nagoya, 464-8601 Japan; ^6^Graduate School of Bioresources, Mie University, Tsu, 514-8507 Japan; ^7^Division of Natural Science, Osaka Kyoiku University, Kashiwara, 582-8582 Japan; ^8^Graduate School of Life and Environmental Sciences, Osaka Prefecture University, Sakai, 599-8531 Japan; ^9^Graduate School of Environmental and Life Science, Okayama University, Okayama, 700-8530 Japan; ^10^Graduate School of Life Sciences, Tohoku University, Sendai, 980-8577 Japan; ^11^These authors contributed equally to this work.

**Keywords:** Correspondence analysis, Database, Gene expression network, Manual curation, Natural language processing (NLP), Omics

## Abstract

Comprehensive integration of large-scale omics resources such as genomes, transcriptomes and metabolomes will provide deeper insights into broader aspects of molecular biology. For better understanding of plant biology, we aim to construct a next-generation sequencing (NGS)-derived gene expression network (GEN) repository for a broad range of plant species. So far we have incorporated information about 745 high-quality mRNA sequencing (mRNA-Seq) samples from eight plant species (*Arabidopsis thaliana*, *Oryza sativa*, *Solanum lycopersicum*, *Sorghum bicolor*, *Vitis vinifera*, *Solanum tuberosum*, *Medicago truncatula* and *Glycine max*) from the public short read archive, digitally profiled the entire set of gene expression profiles, and drawn GENs by using correspondence analysis (CA) to take advantage of gene expression similarities. In order to understand the evolutionary significance of the GENs from multiple species, they were linked according to the orthology of each node (gene) among species. In addition to other gene expression information, functional annotation of the genes will facilitate biological comprehension. Currently we are improving the given gene annotations with natural language processing (NLP) techniques and manual curation. Here we introduce the current status of our analyses and the web database, PODC (Plant Omics Data Center; http://bioinf.mind.meiji.ac.jp/podc/), now open to the public, providing GENs, functional annotations and additional comprehensive omics resources.

## Introduction

The plant sciences have a unique and distinctive position because of their relationship to human food, culture and civilization. In particular, because of the world population explosion and fossil fuel exhaustion, the plant sciences are thought to be critically related to the future of human culture in the context of food security, biofuel production and sustainability. Hence in this big data era, maintenance of more comprehensive research resources, particularly for pan-omics data repositories, is required (Obayashi and Yano 2014). To this end, we maintain the OryzaExpress (gene expression and annotation database for rice) (Hamada et al. 2011), TOMATOMICS (multiomics database for tomato) (Kobayashi et al. 2014) and other species-specific crop databases.

With the availability of next-generation sequencing (NGS), the distinctiveness of the plant sciences is not only unyielding, but also taking on growing importance. The progress of plant genomics is particularly prominent in this century. Currently, not only typical model plants as represented by Arabidopsis (Arabidopsis Genome Initiative 2000) or rice (International Rice Genome Sequencing Project 2005), but also non-model genome sequences have been deciphered and published (Garcia-Mas et al. 2012, Chagne et al. 2014, Schmutz et al. 2014), and corresponding genome-related databases have been constructed (Ohyanagi et al. 2006, Tanaka et al. 2008, Bombarely et al. 2011, Goodstein et al. 2012, Lamesch et al. 2012, Sakai et al. 2013).

Among multilayer plant omics information, the transcriptome, which inscribes the profile of the total content and quantity of mRNA molecules, has been understood as an invaluable clue to predict gene functions based on gene expression similarity or to disclose the hidden molecular mechanisms behind the gene expression regulatory system, i.e. transcription factors, *cis*-regulatory elements and small RNAs. Actually, large-scale transcriptome analyses and database construction have been conducted by taking advantage of microarray technologies (Hamada et al. 2011, Mutwil et al. 2011, Sato et al. 2013a, Sato et al. 2013b, Obayashi et al. 2014).

In recent years, we have focused on the emerging technology of NGS, and have found particularly that mRNA sequencing (mRNA-Seq), an application focusing on the layer of the transcriptome, is tremendously useful. In the plant sciences, third parties have already been analyzing and accumulating mRNA-Seq information, and opening them up to the public domain (Li et al. 2013, Postnikova et al. 2013, Ramilowski et al. 2013, Van Moerkercke et al. 2013, Liu et al. 2014). While a few of the previously mentioned gene expression databases include some mRNA-Seq data sets (Mutwil et al. 2011, Obayashi et al. 2014), we now aim to analyze comprehensively information on mRNA-Seq across a broad range of species, predict gene expression networks (GENs) using the expression profiles derived from the mRNA-Seq analysis outcomes, and establish them as a core resource of a pan-omics database. The GENs of multiple species should not be isolated from each other (Mutwil et al. 2011, Heyndrickx and Vandepoele 2012), so we are trying to connect them according to the orthologous relationships of compound genes, enabling the evolutionary comprehension of the total network. In addition, we are employing natural language processing (NLP) and manual curation as an advanced option with the aim of enhancing the quality of gene annotations. Specifically the PubMed (http://www.ncbi.nlm.nih.gov/pubmed) sentences were interpreted and summarized with proprietary NLP tools, and the relationships between two protein identifiers or between a protein identifier and a phenomenon were extracted. Then the co-occurrence relationships are manually curated and determined as the final NLP outcome.

Our goal is to establish a pan-omics database, the Plant Omics Data Center (PODC; http://bioinf.mind.meiji.ac.jp/podc/), that includes core gene expression information. Here we introduce the current status of the PODC and discuss the future direction of this database.

## Results

### GEN analysis

The GEN is an ideal technique for grasping similarities of expression profiles among genes simultaneously. By taking advantage of the correspondence analysis (CA) algorithm, we have developed a statistical method to analyze large-scale gene expression profiles to construct GENs (see the Materials and Methods). This method classifies genes according to similarities in gene expression profiles.

For construction of the PODC, we calculated similarities of gene expression profiles with mRNA-Seq expression analysis results (see the Materials and Methods) and the CA algorithm. According to a heuristic manual validation of network adequacy, currently we have defined the top 0.1% of gene pairs in expression similarities as being similarly expressed gene pairs (*Arabidopsis thaliana*, 622,462 pairs; *Oryza sativa*, 983,974 pairs; *Solanum lycopersicum*, 512,368 pairs; *Sorghum bicolor*, 763,018 pairs; *Vitis vinifera*, 1,442,892 pairs; *Solanum tuberosum*, 1,386,466 pairs; *Medicago truncatula*, 1,445,827 pairs; *Glycine max*, 3,837,387 pairs) and stored this information in the database. Currently the threshold (0.1%) for significant similarity is a fixed value in the system, but is planned to be a variable value.

### Orthology detection among multiple plant species

By the means of the OrthoMCL procedure described in the Materials and Methods, 3,780,141 orthologous gene pairs among the eight species were detected, stored in the database and employed to connect interspecies GENs.

### NLP and manual curation

Currently we have been focusing on plant reproduction terminology, and gathered the PubMed papers by keyword search (Table 1). Then a total of >28,000 papers were subjected to NLP and manual curation (see the Materials and Methods). As a consequence, the number of relationships we obtained was 1,772 in *A. thaliana*, 92 in *O. sativa*, 119 in *S. lycopersicum*, two in *S. bicolor*, none in *V. vinifera*, 11 in *S. tuberosum*, one in *M. truncatula* and six in *G. max.* The NLP relationships are currently stored in the database as text, but will be graphically shown in the GEN viewer (see Database Functions and Web Interface) in the near future.

**Table 1:** The number of PubMed papers for NLP and manual curation

Keyword*^a^*	*Arabidopsis thaliana*	*Oryza sativa*	*Solanum lycopersicum*	*Sorghum bicolor*	*Vitis vinifera*	*Medicago truncatula*	*Solanum tuberosum*	*Glycine max*
Reproduction	367	213	23	17	27	11	95	110
Fertilization	183	185	18	12	11	15	34	28
Flowering	1,303	515	36	36	44	38	50	123
Pistil	48	33	3	0	0	0	13	1
Heading	20	277	0	1	0	0	–	–
Pollen	729	381	25	13	10	8	60	42
Embryo	557	290	4	15	16	35	22	83
Hybrid	738	675	34	34	42	21	128	56
Yield	423	1,185	60	63	64	29	316	369
Meiosis	242	109	4	4	5	3	17	5
Vernalization	147	15	0	0	0	2	–	–
Flower development	172	49	7	0	9	6	4	2
Pollination	137	75	26	9	7	8	9	15
Short-day	127	69	1	4	3	1	26	13
Long-day	126	69	0	0	1	3	14	15
Incompatibility	75	23	3	2	2	0	14	3
Inflorescence	373	97	8	12	17	4	–	3
Endosperm	204	479	5	32	6	9	36	13
Anther	160	190	6	2	4	3	2	2
Fruit	275	170	358	0	442	8	167	50
Sterility	125	249	5	9	1	1	13	20
Flowering/anthesis	1,337	561	49	39	59	–	55	138
Flowering/fertilization	1,444	690	46	49	57	–	84	149
Flowering/flower development	1,445	548	35	37	51	–	54	–
Floral initiation/flower bud initiation/ floral differentiation/flower development/ flower bud differentiation	202	51	8	9	9	–	4	6
Heading/ear emergence	20	280	0	1	0	–	–	–
Seed-setting/fruition / fruit	281	198	359	1	446	–	167	–
Fertilization/syngamy/pollination	291	250	36	21	17	–	41	41
Long-day/short-day	187	89	1	4	3	–	32	17
Crossbreeding/hybridization	856	891	58	50	52	97	264	158
Total	12,594	8,906	1,218	476	1,405	302	1,721	1,462

A list of keywords for plant reproduction processes and the corresponding number of papers in each PubMed search is shown.

*^a^* A solidus (/) indicates search for papers containing either keywords.

PubMed search query (examples): ‘*Arabidopsis thaliana*’ AND ‘reproduction’.

(‘*Arabidopsis thaliana*’ AND ‘flowering’) OR (‘*Arabidopsis thaliana*’ AND ‘anthesis’).

## Database Functions and Web Interface

### How to search the database content

**Fig. 1:**
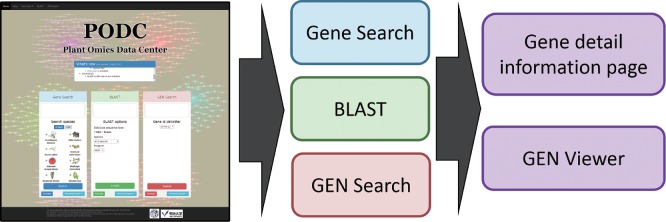
Home page and flowchart of the PODC. A keyword search for gene annotations including NLP relationships (blue pane), a sequence homology search with the BLAST program (green pane) and a GEN search using gene IDs (red pane) are available. In each search result page, the gene detail information page and GEN viewer are hyperlinked.

**Fig. 2:**
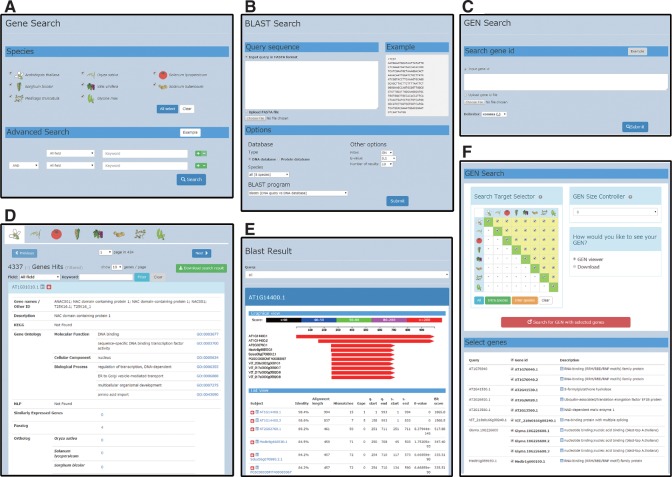
Search query pages (advanced search) and search result pages of the PODC. (A) Gene search query page. (B) BLAST search query page. (C) GEN search query page. (D) Gene search result page. (E) BLAST search result page. (F) GEN search result page. Each search result is also downloadable as a table file.

On the home page of the PODC (http://bioinf.mind.meiji.ac.jp/podc/) (Fig. 1), three quick search functions, a keyword search for gene annotations including NLP relationships (Fig. 1, blue pane), a sequence homology search with the BLAST program (Fig. 1, green pane) and a GEN search using gene IDs (Fig. 1, red pane) are available. For each function, an advanced search page is also implemented (Fig. 2A–C). From each search result page (Fig. 2D–F), particular genes are selected and a corresponding GEN can be drawn. By clicking the plus symbol (icon) on each gene search result page (Fig. 2D, E), users can also create a list of arbitrary genes and draw a GEN for them. Each search result is downloadable as a table file, and detailed information on listed genes is available via designated hyperlinks (Fig. 2D–F).

### Gene detail information

**Fig. 3:**
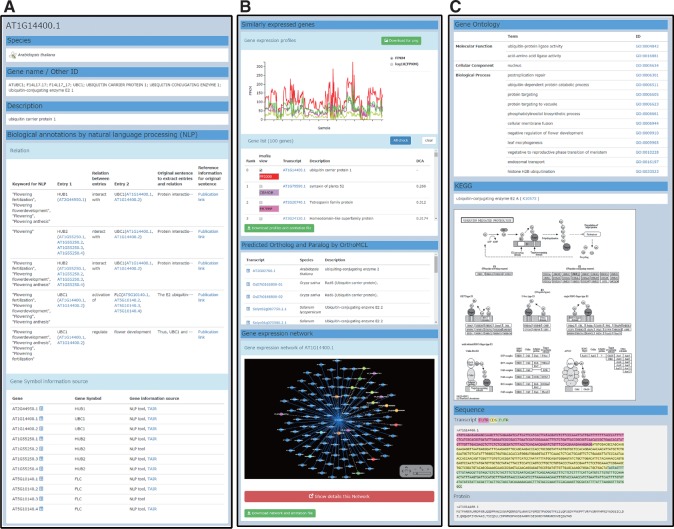
Gene detail information page. Each page has a vertically long layout and contains functional annotations (A), NLP annotations (A), genes having similar expression patterns and their gene expression profile (B), orthologous and paralogous genes (B), the GEN (B), GO annotations (C), KEGG pathway information (C), and DNA and amino acid sequences (C).

The current version of the PODC provides the following data categories on the gene detail information page (Fig. 3): functional annotations (Fig. 3A), NLP annotations (Fig. 3A), genes having similar expression patterns and their gene expression profile (Fig. 3B), orthologous and paralogous genes (Fig. 3B), the GEN (Fig. 3B), gene ontology (GO) annotations (Fig. 3C), KEGG pathway information (Fig. 3C), and DNA and amino acid sequences (Fig. 3C). The profiles of similarly expressed genes can be graphically compared on the page and downloaded (Fig. 3B).

### GEN viewer

**Fig. 4:**
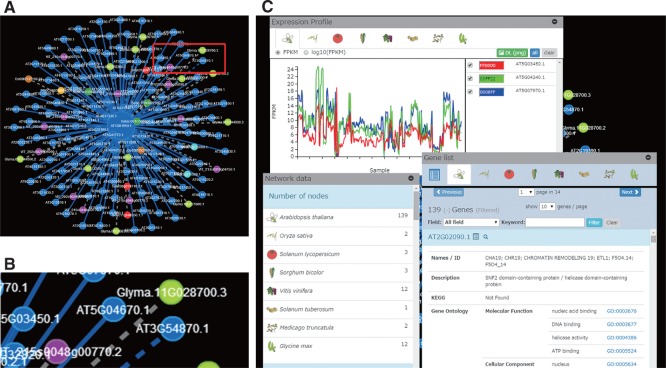
Details in GEN viewer. (A) An interspecies network with genes from multiple species. Each node indicates a gene, each solid edge means a relationship (a similarly expressed gene pair) and each dashed edge represents an orthologous or paralogous relationship. Some of those genes are orthologous to the centered Arabidopsis gene (gray dashed edges). (B) Zoomed-in view of the red box in (A). The blue dashed edge represents a paralogous relationship between two Arabidopsis genes. (C) Detailed information pages including for gene expression profiles, network members and gene annotations.

Visualization of GEN as a network graphic makes it easier to understand the relationships among multiple genes and the characteristics of gene clusters. The web interface for GEN was constructed with Cytoscape Web (http://cytoscapeweb.cytoscape.org/) (Lopes et al. 2010) (Fig. 4A), a graphic network visualization tool. In terms of network representations, each node indicates a gene, and each edge means a relationship (Fig. 4A). In the case of the PODC, each solid edge indicates a similarly expressed gene pair, and each dashed edge represents an orthologous or paralogous relationship (Fig. 4A, B). The colors of nodes and edges correspond to the eight plant species and orthologous relationship. Our GEN viewer allows zooming in and out, panning, and moving nodes and edges with drag-and-drop functionality.

The number of simultaneously visualizable nodes is about 1,000–2,000 (dependent on client PC specification). A brief annotation of each gene pops up by scrolling a mouse cursor over the node. Detailed information including gene expression profile, orthologous genes and NLP annotations are shown by clicking or selecting particular nodes (Fig. 4C). Each gene in GEN is accessible with a keyword search.

When searched genes (nodes) are selected, the node border color changes. GENs can be interactively expanded by every single path from a selected gene, or selected genes can be removed. The number of nodes for each species and number of edges for types of relationship within the GEN are shown (Fig. 4C). Information on functional annotations, sequences and expression profiles of genes within each GEN are downloadable. The GEN data are also downloadable in SIF (simple interaction format) or as an image (PNG format). The SIF file is portable to Cytoscape (Shannon et al. 2003).

To provide an example of the GEN, *A. thaliana* genes encoding enzymes functioning in the photosynthetic Calvin–Benson cycle (CBC) were obtained from the Plant Metabolic Network (http://www.plantcyc.org/; Chae et al. 2014) and used to draw GENs for the eight species. As expected, the resulting GENs demonstrated expression networks of the CBC genes in the species (Supplementary Fig. S1A). While the GENs were varied across species, some relationships of similarly expressed genes were conserved among multiple species such as between a sedoheptulose-1,7-bisphosphatase (SBPase) gene and a fructose-1,6-bisphosphatase (FBPase) gene in *A. thaliana*, *S. tuberosum* and *M. truncatula*. More mRNA-Seq data are being accumulated than those of microarray platforms in recent years, and the sensitivity and accuracy of PODC GEN detection will be improved along with obtaining more sample variations.

The *A. thaliana* GEN of the CBC was further evaluated by comparison with one drawn in another web tool, ATTED-II, which uses microarray data (Obayashi et al. 2014). GENs drawn in both web tools are summarized in Supplementary Fig. S1B. Again, an SBPase gene (AT3G55800) and an FBPase gene (AT3G54050, known as high cyclic electron flow 1) were found to be similarly expressed in ATTED-II as well as in the PODC. SBPase and FBPase are considered to be key steps in regulating carbon flow of the CBC (Tamoi et al. 2005, Liu et al. 2012), and their enzymatic activities are regulated by light condition via thioredoxin (Michelet et al. 2013). Given that the gene expression similarity of SBPase and FBPase is conserved among species, we can hypothesize that co-ordinated fundamental regulation of gene expression of SBPase and FBPase is important as an understructure sustaining precise modulation of the CBC functions. A relationship between AT3G12780 (phosphoglycerate kinase 1) and AT1G42970 (glyceraldehyde-3-phosphate B subunit) was also found in both tools.

Several similarly expressed gene pairs were found only in one of the two tools. There are many potential causes of such differences: different platform (NGS and microarray), different sample set and different method to detect gene expression similarities (CA analysis and Pearson’s correlation coefficient). Because of the complexity, it is fairly difficult to identify the actual factor making the differences. However, in terms of the expression similarity among ribulose-1,5-bisphosphate carboxylase/oxygenase small subunit (RbcS) genes (AT1G67090, AT5G38410, AT5G38420 and AT5G38430), the primary reason why the relationship is not found in ATTED-II but is found in the PODC is clear: probes on the microarray cannot separate the family genes because of the high identity in nucleotide sequence, but mRNA-Seq can do it. This exemplifies an advantage of employing mRNA-Seq data to construct GENs. In principle, mRNA-Seq can quantify the expression levels of all gene models separately, unless those sequences are 100% identical. Moreover, we believe that the future accumulation of mRNA-Seq samples will enhance the advantages of the PODC.

## Conclusion and Future Direction

Here we introduced the PODC, a web repository for NGS transcriptomes and GENs with an interactive network viewer. Compared with existing GEN databases (Mutwil et al. 2011, Obayashi et al. 2014), the content depth of NGS mRNA-Seq data in our PODC seems without equal. In addition, we are taking advantage of the state-of-the-art NLP technique for cost-effective accumulation of manually curated plant annotations. We believe that these multiple enrichments of data content make our database unique and invaluable in the plant sciences.

We are still enhancing the data content and improving the web interface. As for future plans, we aim to add more plant species; not only model crops, but also minor and non-model plant species. We would also consider incorporating mRNA-Seq reads produced by non-Illumina platforms. In addition, we plan to add more NLP keywords for biotic/abiotic stresses and other critical plant biology terms. Moreover, we are implementing a prediction program for *cis*-regulatory elements (manuscript in preparation) that are strongly related to GENs in terms of hidden molecular mechanisms for control of gene expression.

We are mainly focusing on the transcriptome, but we plan to broaden the content of the database, i.e. to proteomes, metabolomes and phenomes. We believe that the GEN information in the PODC will become its core information, and make it easy to navigate throughout every plant omics layer.

## Materials and Methods

### Gene expression data from public data repositories

Illumina mRNA-Seq reads for eight plant species (*A. thaliana*, *O. sativa*, *S. bicolor*, *S. lycopersicum*, *V. vinifera*, *M. truncatula*, *S. tuberosum* and *G. max*) were downloaded from the NCBI SRA database (http://www.ncbi.nlm.nih.gov/sra) (NCBI Resource Coordinators 2014). In total, 1,700 samples (*A. thaliana*, 709 samples; *O. sativa*, 221 samples; *S. lycopersicum*, 199 samples; *S. bicolor*, 37 samples; *V. vinifera*, 41 samples; *S. tuberosum*, 114 samples; *M. truncatula*, 34 samples; *G. max*, 345 samples) were initially downloaded along with a variety of information about biological and experimental conditions, such as time courses, stress treatments, growth stages, organs, transformed plants and mutant lines.

### mRNA-Seq analysis

To construct a GEN, transcriptome profiling and quantification of gene expression levels are comprehensively performed by mapping the mRNA-Seq reads to reference genome sequences. We downloaded eight reference genomes (*A. thaliana*, *O. sativa*, *S. lycopersicum*, *S. bicolor*, *V. vinifera*, *S. tuberosum*, *M. truncatula* and *G. max*) from the Arabidopsis Information Resource (TAIR) (http://www.arabidopsis.org/) (Lamesch et al. 2012), the Rice Annotation Project Database (RAP-DB) (http://rapdb.dna.affrc.go.jp/) (Ohyanagi et al. 2006, Tanaka et al. 2008, Sakai et al. 2013), the Sol Genome Network (http://solgenomics.net/) (Bombarely et al. 2011), the Grape Genome Database (http://genomes.cribi.unipd.it/grape/) (Vitulo et al. 2014) and the Phytozome (http://www.phytozome.net/) (Goodstein et al. 2012). After quality control by FastQC (http://www.bioinformatics.babraham.ac.uk/projects/fastqc/), we trimmed adaptor sequences by cutadapt (https://code.google.com/p/cutadapt/) and filtered out the low-quality reads by an empirically optimized custom Perl script. Its filters are as follows: (i) both ends of each read should have QV ≥10 (if it is not, the end base will be trimmed away until QV ≥10 is exposed); (ii) each read should have average QV ≥17; (iii) final length of each read should be ≥20 bp; (iv) each read should have low-quality positions (QV <10) no more than 10% of final length; and (v) each read should not contain any N bases. Consequently, 745 samples (*A. thaliana*, 264 samples; *O. sativa*, 73 samples; *S. lycopersicum*, 120 samples, *S. bicolor*, 37 samples; *V. vinifera*, 36 samples; *S. tuberosum*, 34 samples; *M. truncatula*, 33 samples; *G. max*, 148 samples) remained and were further analyzed. These mRNA-Seq reads were then aligned to each reference genome by TopHat (Kim et al. 2013) and gene expression was quantified by Cufflinks (Trapnell et al. 2013). The publicly available gene models (see ‘Gene annotations’) were employed as TopHat and Cufflinks reference annotations with the -G option. Except for this, TopHat and Cufflinks were run with default parameters.

### GEN analysis

We evaluated similarities in gene expression profiles of each gene by CA as described in our previous reports (Yano et al. 2006, Hamada et al. 2011). Conceptually CA summarizes a gene expression data matrix into a lower dimensional space. For each gene and sample, co-ordinates in the low-dimensional space are provided. With these co-ordinates, genes can be plotted in a three-dimensional space. Theoretically, genes with similar expression profiles are closely related. Therefore, the distance between genes in the low-dimensional space indicates similarity in gene expression profiles.

The gene expression profiles determined by mRNA-Seq analysis were subjected to the CA procedure (Yano et al. 2006, Hamada et al. 2011). Then the deduced similarity relationships were inspected with the GUI software tool called CA Plot Viewer (http://bioinf.mind.meiji.ac.jp/lab/), and employed as gene expression similarities in PODC.

### Orthology detection among multiple plant species

Orthologous gene pair detection among the eight plant species was performed by employing the OrthoMCL algorithm (http://orthomcl.org/orthomcl/) (Li et al. 2003) by default parameters. First, deduced protein sequences derived from all gene nucleotide sequences were quality controlled by a filter command in OrthoMCL (orthomclFilterFasta 10 20). Secondly, the cleaned protein sequences were concatenated to a single FASTA file, and employed to detect BLASTP (Altschul et al. 1997) similarities among the entire protein sequence set (blastall -p blastp -m 8 -F ‘m S’ -v 100000 -b 100000 -z 414453 -e 1e-5 -a 20). Then OrthoMCL commands orthomclLoadBlast and orthomclDumpPairsFiles were run with a configuration (percentMatchCutoff=50, evalueExponentCutoff=-5) on the BLASTP results in order to find potential inparalogous, orthologous and co-orthologous pairs. Finally the MCL clusters were determined with an OrthoMCL command (mcl —abc -I 1.5).

### NLP and manual curation

Functional annotation strategies are mainly based on sequence similarity searches against functionally determined genes. However, more accurate functional annotation would be based on literature information with so-called manual curation. Manual curation requires the curators to have particular skills in interpreting the literature, and it is quite time consuming. The NLP technique is thought to be a breakthrough in this process. It has the potential to gather information faster than manual curation, but still has the technical problem regarding the accuracy of its results. Here we aim to combine NLP and manual curation, i.e. first we input a massive amount of literature information into the NLP program, then we validated the NLP results manually. With this strategy, we believe that higher quality functional annotations will be generated with a relatively small amount of manual effort.

As a rough idea, our NLP tools (MedScan and PathwayStudio, http://www.elsevier.com/online-tools/pathway-studio/about/pathway-studio-plant) (Novichkova et al. 2003, Yuryev et al. 2006) co-ordinately interpret and summarize PubMed sentences with a dictionary based on *A. thaliana*, and the outcome contains relationships between two protein identifiers or between a protein identifier and a phenomenon. Since the relationships are based on *A. thaliana* gene nomenclature, we have to convert the Arabidopsis gene IDs or gene symbols into those of the other seven plant species. To convert the IDs, orthologous relationships in UniProt (http://www.uniprot.org/), TAIR (http://www.arabidopsis.org/), RAP-DB (http://rapdb.dna.affrc.go.jp/), SGN (http://solgenomics.net/) and BioMart (http://www.biomart.org/) (Kasprzyk 2011) are manually employed. Simultaneously, the co-occurrence relationships are manually extracted and curated as the final NLP outcome.

More precisely, particular terms (Table 1) were firstly searched on PubMed (http://www.ncbi.nlm.nih.gov/pubmed), and the results were saved in XML format. Secondly, the results in XML files were processed by the MedScan program and each pair of related terms (protein, small molecule, complex, cell process, cell object, disease, functional class and treatment) in a PubMed sentence was automatically extracted. Then the extracted relationships were manually inspected and relationships concerning proteins were selected (by taking advantage of MedScan filter function); simultaneously the orthologous relationships in UniProt (http://www.uniprot.org/), TAIR (http://www.arabidopsis.org/), RAP-DB (http://rapdb.dna.affrc.go.jp/), SGN (http://solgenomics.net/) and BioMart (http://www.biomart.org/) (Kasprzyk 2011) were manually employed to convert the IDs. Finally the selected relationships were subjected to PathwayStudio (by MedScan Send to PathwayStudio function) in order to summarize the final list of NLP annotations.

### Gene annotations

For each gene, the functional descriptions, GO terms and DNA/amino acid sequences were incorporated from TAIR (http://www.arabidopsis.org/) (Lamesch et al. 2012), RAP-DB (http://rapdb.dna.affrc.go.jp/) (Ohyanagi et al. 2006, Tanaka et al. 2008, Sakai et al. 2013), the Sol Genome Network (http://solgenomics.net/) (Bombarely et al. 2011), the Grape Genome Database (http://genomes.cribi.unipd.it/grape/) (Vitulo et al. 2014) or the Phytozome (http://www.phytozome.net/) (Goodstein et al. 2012), if available. Each gene was also described with rich annotations represented by NLP and manually curated information or KEGG pathways (Kanehisa et al. 2014). Each of them was hyperlinked to the original source. In addition, those gene models were employed as TopHat and Cufflinks reference annotations (see ‘mRNA-Seq analysis’).

### System architecture and software

The PODC was implemented on a UNIX server with CentOS version 5, Apache web server and MySQL Database server. PHP version 5 was employed as a server-side scripting language. JavaScript was adopted to implement client-side rich applications. As for JavaScript libraries, jQuery (http://jquery.com), jQuery UI (http://jqueryui.com), Bootstrap (http://getbootstrap.com), D3 (http://d3js.org) and Cytoscape Web (http://cytoscapeweb.cytoscape.org) were employed. Other conventional utilities for UNIX computing were appropriately installed on the server if necessary. All of the PODC resources are stored in the server and available through HTTP access.

A GUI software tool called CA Plot Viewer (http://bioinf.mind.meiji.ac.jp/lab/) was employed in the manual inspection step in GEN analysis.

## Supplementary data

Supplementary data are available at PCP online.

## Funding

This work is supported by the Japan Society for the Promotion of Science (JSPS) [Grants-in-Aid for Scientific Research on Innovative Areas (No. 26113716 to K.Y., No. 23113006 to G.S., No. 23113005 to M.M., No. 23113001 to G.S. and M.M.), Scientific Research (A) (No. 23248005 to K.A., No. 25252001 to M.W.), Scientific Research (B) (No. 25292005 to K.S., No. 24380023 to Y.K.) and Scientific Research (C) (No. 25450515 to G.S.); the Ministry of Education, Culture, Sports, Science and Technology of Japan (MEXT) [Supported Program for the Strategic Research Foundation at Private Universities (2014–2018)]; Meiji University [Research Funding for Computational Software Supporting Program].

## Disclosures

The authors have no conflicts of interest to declare.

## Supplementary Material

Supplementary Data

## Abbreviations

CA: correspondence analysis
CBC: Calvin–Benson cycle
FBPase: fructose-1,6-bisphosphatase
GEN: gene expression network
GO: gene ontology
mRNA-Seq: mRNA sequencing
NGS: next-generation sequencing
NLP: natural language processing
PODC: Plant Omics Data Center
SBPase: sedoheptulose-1,7-bisphosphatase
