# Supplementary material for: Plant Omics Data Center: An Integrated Web Repository for Interspecies Gene Expression Networks with NLP-Based Curation
Source: Plant Cell Physiol. 2014 Dec 11;56(1):e9. doi: 10.1093/pcp/pcu188 (PMC4301748; doi:10.1093/pcp/pcu188)
Supplement: Supplementary Data [file supp_56_1_e9__index.html]

Plant Omics Data Center: An Integrated Web Repository for Interspecies Gene Expression Networks with NLP-Based Curation — Plant Omics Data Center: An Integrated Web Repository for Interspecies Gene Expression Networks with NLP-Based Curation — Supplementary Data 

# Plant Omics Data Center: An Integrated Web Repository for Interspecies Gene Expression Networks with NLP-Based Curation

## Supplementary Data

files

**Files in this Data Supplement:**

- Supplementary Data - pdf file
