# Supplementary material for: Plant Omics Data Center: An Integrated Web Repository for Interspecies Gene Expression Networks with NLP-Based Curation
Source: Plant Cell Physiol. 2014 Dec 11;56(1):e9. doi: 10.1093/pcp/pcu188 (PMC4301748; doi:10.1093/pcp/pcu188)
Supplement: Supplementary Data [file supp_pcu188_pcp-2014-e-00412-File006.pdf]

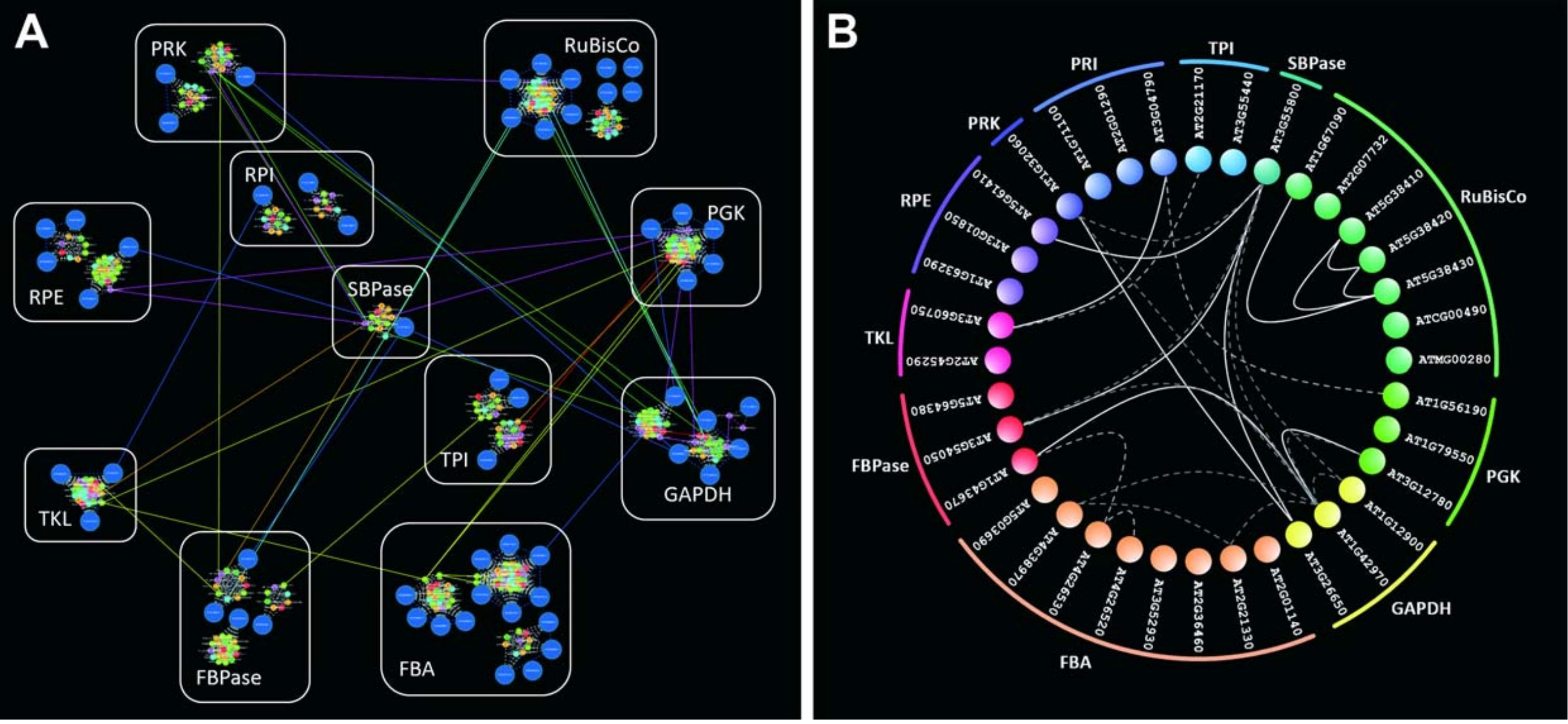
A
PRK
RuBisCo
RPI
PGK
RPE
SBPase
TPI
GAPDH
TKL
FBPase
FBA
B
PRI
TPI
SBPase
PRK
RPE
RuBisCo
TKL
FBPase
PGK
GAPDH
FBA
AT1G71100
AT2G01290
AT3G04790
AT2G21170
AT3G55440
AT3G55800
AT1G67090
AT2G07732
AT5G38410
AT5G38420
AT5G38430
ATCG00490
ATMG00280
AT1G56190
AT1G79550
AT3G12780
AT1G12900
AT1G42970
AT3G26650
AT2G01140
AT2G21330
AT2G36460
AT3G52930
AT4G26520
AT4G26530
AT4G38970
AT5G03690
AT1G43670
AT3G54050
AT5G64380
AT2G45290
AT3G60750
AT1G63290
AT3G01850
AT5G61410
AT1G32060

Supplemental Figure 1. GEN for photosynthesis related genes. (A) The gene components in the Calvin-Benson-Bassham cycle were searched in PODC and the GEN was drawn. (B) Comparison of Calvin-Benson cycle GENs generated in PODC and ATTED-II. Gene pairs found to be similarly expressed in PODC and ATTED-II are connected with solid lines and dashed lines, respectively. The components of cycle are as follows; FBA (fructose-bisphosphate aldolase), FBPase (fructose-1,6-bisphosphatase), GAPDH (glyceraldehyde-3-phosphate dehydrogenase), PGK (phosphoglycerate kinase), PRK (phosphoribulokinase), RPE (ribulose-phosphate 3-epimerase), RPI (ribose-5-phosphate isomerase), Rubisco (ribulose-1,5-bisphosphate carboxylase/oxygenase), SBPase (sedoheptulose-1,7-bisphosphatase), TKL (transketolase), and TPI (triose-phosphate isomerase).

SRA Run accession	Species	Is qualified?
SRR515181	Arabidopsis thaliana	yes
SRR515180	Arabidopsis thaliana	yes
ERR229835	Arabidopsis thaliana	no
ERR229837	Arabidopsis thaliana	no
ERR229851	Arabidopsis thaliana	no
ERR229848	Arabidopsis thaliana	no
ERR229849	Arabidopsis thaliana	no
ERR229845	Arabidopsis thaliana	no
ERR229855	Arabidopsis thaliana	no
ERR229826	Arabidopsis thaliana	no
ERR229853	Arabidopsis thaliana	no
ERR229857	Arabidopsis thaliana	yes
ERR229839	Arabidopsis thaliana	no
ERR229846	Arabidopsis thaliana	yes
ERR229832	Arabidopsis thaliana	yes
ERR229829	Arabidopsis thaliana	no
ERR229838	Arabidopsis thaliana	yes
ERR229843	Arabidopsis thaliana	no
ERR229831	Arabidopsis thaliana	yes
ERR229833	Arabidopsis thaliana	no
ERR229858	Arabidopsis thaliana	yes
ERR229828	Arabidopsis thaliana	no
ERR229836	Arabidopsis thaliana	yes
ERR229842	Arabidopsis thaliana	no
ERR229840	Arabidopsis thaliana	no
ERR229847	Arabidopsis thaliana	yes
ERR229834	Arabidopsis thaliana	no
ERR229854	Arabidopsis thaliana	no
ERR229852	Arabidopsis thaliana	yes
ERR229830	Arabidopsis thaliana	no
ERR229844	Arabidopsis thaliana	no
ERR229827	Arabidopsis thaliana	no
ERR229856	Arabidopsis thaliana	no
ERR229850	Arabidopsis thaliana	no
ERR229841	Arabidopsis thaliana	no
ERR274309	Arabidopsis thaliana	yes
ERR274310	Arabidopsis thaliana	no
ERR274308	Arabidopsis thaliana	yes
ERR274311	Arabidopsis thaliana	no
SRR764885	Arabidopsis thaliana	yes
SRR764886	Arabidopsis thaliana	yes
SRR764887	Arabidopsis thaliana	yes
SRR791229	Arabidopsis thaliana	no
SRR791230	Arabidopsis thaliana	no
SRR791231	Arabidopsis thaliana	no

SRA Run accession	Species	Is qualified?
SRR791232	Arabidopsis thaliana	no
SRR800644	Arabidopsis thaliana	no
SRR800645	Arabidopsis thaliana	no
SRR800753	Arabidopsis thaliana	no
SRR800754	Arabidopsis thaliana	no
SRR390305	Arabidopsis thaliana	no
SRR390310	Arabidopsis thaliana	no
SRR390314	Arabidopsis thaliana	no
SRR391051	Arabidopsis thaliana	yes
SRR391052	Arabidopsis thaliana	yes
SRR837535	Arabidopsis thaliana	no
SRR837536	Arabidopsis thaliana	no
SRR847502	Arabidopsis thaliana	no
SRR847501	Arabidopsis thaliana	no
SRR847503	Arabidopsis thaliana	no
SRR847504	Arabidopsis thaliana	no
SRR847505	Arabidopsis thaliana	no
SRR847506	Arabidopsis thaliana	no
SRR641216	Arabidopsis thaliana	no
SRR641218	Arabidopsis thaliana	no
SRR641217	Arabidopsis thaliana	no
SRR641219	Arabidopsis thaliana	no
SRR641220	Arabidopsis thaliana	no
SRR641221	Arabidopsis thaliana	no
SRR641222	Arabidopsis thaliana	no
SRR641223	Arabidopsis thaliana	no
SRR641224	Arabidopsis thaliana	no
SRR641225	Arabidopsis thaliana	no
SRR641226	Arabidopsis thaliana	no
SRR641227	Arabidopsis thaliana	no
SRR641228	Arabidopsis thaliana	no
SRR641229	Arabidopsis thaliana	no
SRR641230	Arabidopsis thaliana	no
SRR641231	Arabidopsis thaliana	no
SRR641232	Arabidopsis thaliana	no
SRR641233	Arabidopsis thaliana	no
SRR641234	Arabidopsis thaliana	no
SRR641235	Arabidopsis thaliana	no
SRR641236	Arabidopsis thaliana	no
SRR641237	Arabidopsis thaliana	no
SRR641238	Arabidopsis thaliana	no
SRR641239	Arabidopsis thaliana	no
SRR641240	Arabidopsis thaliana	no
SRR641241	Arabidopsis thaliana	no
SRR641242	Arabidopsis thaliana	no

SRA Run accession	Species	Is qualified?
SRR641243	Arabidopsis thaliana	no
SRR641244	Arabidopsis thaliana	no
SRR641245	Arabidopsis thaliana	no
SRR641246	Arabidopsis thaliana	no
SRR641247	Arabidopsis thaliana	no
SRR641248	Arabidopsis thaliana	no
SRR641249	Arabidopsis thaliana	no
SRR641250	Arabidopsis thaliana	no
SRR641251	Arabidopsis thaliana	no
SRR641252	Arabidopsis thaliana	no
SRR641253	Arabidopsis thaliana	no
SRR641254	Arabidopsis thaliana	no
SRR641255	Arabidopsis thaliana	no
SRR641256	Arabidopsis thaliana	no
SRR641257	Arabidopsis thaliana	no
SRR641258	Arabidopsis thaliana	no
SRR641259	Arabidopsis thaliana	no
SRR641260	Arabidopsis thaliana	no
SRR641261	Arabidopsis thaliana	no
SRR641262	Arabidopsis thaliana	no
SRR641263	Arabidopsis thaliana	no
SRR641264	Arabidopsis thaliana	no
SRR641265	Arabidopsis thaliana	no
SRR641266	Arabidopsis thaliana	no
SRR641267	Arabidopsis thaliana	no
SRR641268	Arabidopsis thaliana	no
SRR641269	Arabidopsis thaliana	no
SRR641270	Arabidopsis thaliana	no
SRR641271	Arabidopsis thaliana	no
SRR641272	Arabidopsis thaliana	no
SRR641273	Arabidopsis thaliana	no
SRR641274	Arabidopsis thaliana	no
SRR641275	Arabidopsis thaliana	no
SRR641276	Arabidopsis thaliana	no
SRR641277	Arabidopsis thaliana	no
SRR019182	Arabidopsis thaliana	no
SRR019183	Arabidopsis thaliana	yes
SRR019184	Arabidopsis thaliana	no
SRR545939	Arabidopsis thaliana	no
SRR576327	Arabidopsis thaliana	yes
SRR576328	Arabidopsis thaliana	no
SRR576329	Arabidopsis thaliana	no
SRR578940	Arabidopsis thaliana	yes
SRR578941	Arabidopsis thaliana	no
SRR578942	Arabidopsis thaliana	yes

SRA Run accession	Species	Is qualified?
SRR578943	Arabidopsis thaliana	no
SRR578944	Arabidopsis thaliana	no
SRR578945	Arabidopsis thaliana	yes
SRR578946	Arabidopsis thaliana	yes
SRR578947	Arabidopsis thaliana	yes
SRR578948	Arabidopsis thaliana	yes
SRR584127	Arabidopsis thaliana	yes
SRR584128	Arabidopsis thaliana	yes
SRR584129	Arabidopsis thaliana	yes
SRR584130	Arabidopsis thaliana	yes
SRR584131	Arabidopsis thaliana	yes
SRR584132	Arabidopsis thaliana	yes
SRR584133	Arabidopsis thaliana	yes
SRR584134	Arabidopsis thaliana	yes
SRR584135	Arabidopsis thaliana	yes
SRR584136	Arabidopsis thaliana	yes
SRR584137	Arabidopsis thaliana	yes
SRR609267	Arabidopsis thaliana	yes
SRR609268	Arabidopsis thaliana	no
SRR609269	Arabidopsis thaliana	yes
SRR609270	Arabidopsis thaliana	yes
SRR631034	Arabidopsis thaliana	yes
SRR631035	Arabidopsis thaliana	no
SRR633726	Arabidopsis thaliana	no
SRR633727	Arabidopsis thaliana	no
SRR633728	Arabidopsis thaliana	no
SRR633729	Arabidopsis thaliana	no
SRR633730	Arabidopsis thaliana	no
SRR633731	Arabidopsis thaliana	no
SRR633732	Arabidopsis thaliana	no
SRR633733	Arabidopsis thaliana	no
SRR633734	Arabidopsis thaliana	no
SRR633735	Arabidopsis thaliana	no
SRR633736	Arabidopsis thaliana	no
SRR633737	Arabidopsis thaliana	no
SRR633738	Arabidopsis thaliana	no
SRR633739	Arabidopsis thaliana	no
SRR633740	Arabidopsis thaliana	no
SRR633741	Arabidopsis thaliana	no
SRR634969	Arabidopsis thaliana	no
SRR634970	Arabidopsis thaliana	no
SRR634971	Arabidopsis thaliana	no
SRR634972	Arabidopsis thaliana	no
SRR634973	Arabidopsis thaliana	no
SRR634974	Arabidopsis thaliana	no

SRA Run accession	Species	Is qualified?
SRR641210	Arabidopsis thaliana	no
SRR641211	Arabidopsis thaliana	no
SRR641212	Arabidopsis thaliana	no
SRR641213	Arabidopsis thaliana	no
SRR641214	Arabidopsis thaliana	no
SRR641215	Arabidopsis thaliana	no
SRR609417	Arabidopsis thaliana	no
SRR609418	Arabidopsis thaliana	no
SRR609407	Arabidopsis thaliana	no
SRR609408	Arabidopsis thaliana	no
SRR609410	Arabidopsis thaliana	no
SRR609414	Arabidopsis thaliana	no
SRR609415	Arabidopsis thaliana	no
SRR609416	Arabidopsis thaliana	no
SRR609411	Arabidopsis thaliana	no
SRR609412	Arabidopsis thaliana	no
SRR609413	Arabidopsis thaliana	no
SRR545948	Arabidopsis thaliana	yes
SRR545949	Arabidopsis thaliana	yes
SRR545953	Arabidopsis thaliana	yes
SRR545950	Arabidopsis thaliana	yes
SRR545954	Arabidopsis thaliana	yes
SRR545952	Arabidopsis thaliana	no
SRR518043	Arabidopsis thaliana	yes
SRR518044	Arabidopsis thaliana	yes
SRR520237	Arabidopsis thaliana	yes
SRR520238	Arabidopsis thaliana	yes
SRR520239	Arabidopsis thaliana	yes
SRR520240	Arabidopsis thaliana	yes
SRR520241	Arabidopsis thaliana	yes
SRR520242	Arabidopsis thaliana	yes
SRR520243	Arabidopsis thaliana	yes
SRR520244	Arabidopsis thaliana	yes
SRR520245	Arabidopsis thaliana	yes
SRR520246	Arabidopsis thaliana	yes
SRR520247	Arabidopsis thaliana	yes
SRR520248	Arabidopsis thaliana	yes
SRR525101	Arabidopsis thaliana	no
SRR525102	Arabidopsis thaliana	yes
SRR525103	Arabidopsis thaliana	yes
SRR525104	Arabidopsis thaliana	no
SRR525105	Arabidopsis thaliana	yes
SRR525106	Arabidopsis thaliana	yes
SRR525107	Arabidopsis thaliana	yes
SRR525108	Arabidopsis thaliana	yes

SRA Run accession	Species	Is qualified?
SRR525109	Arabidopsis thaliana	no
SRR525110	Arabidopsis thaliana	yes
SRR525111	Arabidopsis thaliana	yes
SRR525112	Arabidopsis thaliana	yes
SRR525113	Arabidopsis thaliana	no
SRR525114	Arabidopsis thaliana	yes
SRR525115	Arabidopsis thaliana	yes
SRR525116	Arabidopsis thaliana	no
SRR525117	Arabidopsis thaliana	yes
SRR525118	Arabidopsis thaliana	yes
SRR525119	Arabidopsis thaliana	yes
SRR525120	Arabidopsis thaliana	yes
SRR525121	Arabidopsis thaliana	yes
SRR525122	Arabidopsis thaliana	yes
SRR525123	Arabidopsis thaliana	no
SRR525124	Arabidopsis thaliana	yes
SRR525125	Arabidopsis thaliana	yes
SRR525126	Arabidopsis thaliana	no
SRR525127	Arabidopsis thaliana	no
SRR525128	Arabidopsis thaliana	yes
SRR525129	Arabidopsis thaliana	yes
SRR525130	Arabidopsis thaliana	yes
SRR525131	Arabidopsis thaliana	yes
SRR525132	Arabidopsis thaliana	no
SRR525133	Arabidopsis thaliana	yes
SRR525134	Arabidopsis thaliana	yes
SRR525135	Arabidopsis thaliana	no
SRR525136	Arabidopsis thaliana	no
SRR525137	Arabidopsis thaliana	yes
SRR525138	Arabidopsis thaliana	yes
SRR525139	Arabidopsis thaliana	no
SRR525140	Arabidopsis thaliana	yes
SRR525141	Arabidopsis thaliana	no
SRR525142	Arabidopsis thaliana	yes
SRR525143	Arabidopsis thaliana	yes
SRR525144	Arabidopsis thaliana	yes
SRR525145	Arabidopsis thaliana	yes
SRR525146	Arabidopsis thaliana	no
SRR525147	Arabidopsis thaliana	yes
SRR525148	Arabidopsis thaliana	yes
SRR525149	Arabidopsis thaliana	yes
SRR525150	Arabidopsis thaliana	yes
SRR525151	Arabidopsis thaliana	no
SRR525152	Arabidopsis thaliana	no
SRR525153	Arabidopsis thaliana	yes

SRA Run accession	Species	Is qualified?
SRR525154	Arabidopsis thaliana	no
SRR525155	Arabidopsis thaliana	no
SRR525156	Arabidopsis thaliana	no
SRR525157	Arabidopsis thaliana	yes
SRR525158	Arabidopsis thaliana	no
SRR525159	Arabidopsis thaliana	yes
SRR525160	Arabidopsis thaliana	no
SRR525161	Arabidopsis thaliana	yes
SRR525162	Arabidopsis thaliana	no
SRR525163	Arabidopsis thaliana	yes
SRR525164	Arabidopsis thaliana	yes
SRR525165	Arabidopsis thaliana	yes
SRR525166	Arabidopsis thaliana	yes
SRR525168	Arabidopsis thaliana	no
SRR525170	Arabidopsis thaliana	yes
SRR544881	Arabidopsis thaliana	no
SRR546147	Arabidopsis thaliana	no
SRR546148	Arabidopsis thaliana	no
SRR546149	Arabidopsis thaliana	no
SRR546150	Arabidopsis thaliana	no
SRR546151	Arabidopsis thaliana	no
SRR546152	Arabidopsis thaliana	no
SRR546153	Arabidopsis thaliana	no
SRR546154	Arabidopsis thaliana	no
SRR837534	Arabidopsis thaliana	no
SRR515195	Arabidopsis thaliana	yes
SRR515176	Arabidopsis thaliana	yes
SRR515177	Arabidopsis thaliana	yes
SRR515192	Arabidopsis thaliana	yes
SRR360152	Arabidopsis thaliana	yes
SRR360147	Arabidopsis thaliana	yes
SRR360153	Arabidopsis thaliana	yes
SRR360205	Arabidopsis thaliana	yes
SRR360154	Arabidopsis thaliana	yes
SRR513593	Arabidopsis thaliana	no
SRR513592	Arabidopsis thaliana	no
SRR513594	Arabidopsis thaliana	no
SRR513595	Arabidopsis thaliana	no
SRR513596	Arabidopsis thaliana	yes
SRR513597	Arabidopsis thaliana	no
SRR513730	Arabidopsis thaliana	yes
SRR513729	Arabidopsis thaliana	yes
SRR513732	Arabidopsis thaliana	yes
SRR513731	Arabidopsis thaliana	yes
SRR513741	Arabidopsis thaliana	yes

SRA Run accession	Species	Is qualified?
SRR513742	Arabidopsis thaliana	yes
SRR513743	Arabidopsis thaliana	yes
SRR513744	Arabidopsis thaliana	yes
SRR515178	Arabidopsis thaliana	yes
SRR515179	Arabidopsis thaliana	yes
SRR515193	Arabidopsis thaliana	yes
SRR514997	Arabidopsis thaliana	yes
SRR514998	Arabidopsis thaliana	no
SRR514999	Arabidopsis thaliana	no
SRR501598	Arabidopsis thaliana	no
SRR501599	Arabidopsis thaliana	yes
SRR501604	Arabidopsis thaliana	no
SRR501605	Arabidopsis thaliana	yes
SRR501600	Arabidopsis thaliana	yes
SRR501601	Arabidopsis thaliana	yes
SRR501606	Arabidopsis thaliana	no
SRR501607	Arabidopsis thaliana	yes
SRR501608	Arabidopsis thaliana	no
SRR501609	Arabidopsis thaliana	yes
SRR501602	Arabidopsis thaliana	no
SRR501603	Arabidopsis thaliana	yes
SRR501610	Arabidopsis thaliana	yes
SRR501611	Arabidopsis thaliana	yes
SRR501617	Arabidopsis thaliana	no
SRR501618	Arabidopsis thaliana	no
SRR501612	Arabidopsis thaliana	no
SRR501613	Arabidopsis thaliana	yes
SRR501619	Arabidopsis thaliana	no
SRR501620	Arabidopsis thaliana	yes
SRR501621	Arabidopsis thaliana	no
SRR501622	Arabidopsis thaliana	yes
SRR501614	Arabidopsis thaliana	no
SRR501615	Arabidopsis thaliana	yes
SRR501616	Arabidopsis thaliana	yes
SRR501623	Arabidopsis thaliana	yes
SRR504179	Arabidopsis thaliana	yes
SRR504180	Arabidopsis thaliana	yes
SRR504181	Arabidopsis thaliana	yes
SRR504182	Arabidopsis thaliana	no
SRR504183	Arabidopsis thaliana	no
SRR504184	Arabidopsis thaliana	no
SRR504185	Arabidopsis thaliana	no
SRR504186	Arabidopsis thaliana	no
SRR504187	Arabidopsis thaliana	no
SRR515317	Arabidopsis thaliana	no

SRA Run accession	Species	Is qualified?
SRR515318	Arabidopsis thaliana	no
SRR515319	Arabidopsis thaliana	no
SRR515320	Arabidopsis thaliana	no
SRR515321	Arabidopsis thaliana	no
SRR515322	Arabidopsis thaliana	no
SRR515323	Arabidopsis thaliana	no
SRR515324	Arabidopsis thaliana	no
SRR515325	Arabidopsis thaliana	no
SRR515326	Arabidopsis thaliana	no
SRR515327	Arabidopsis thaliana	no
SRR515328	Arabidopsis thaliana	no
SRR515329	Arabidopsis thaliana	no
SRR515330	Arabidopsis thaliana	no
SRR515331	Arabidopsis thaliana	no
SRR515332	Arabidopsis thaliana	no
SRR515333	Arabidopsis thaliana	no
SRR515334	Arabidopsis thaliana	no
SRR515335	Arabidopsis thaliana	no
SRR515336	Arabidopsis thaliana	no
SRR515337	Arabidopsis thaliana	yes
SRR515338	Arabidopsis thaliana	no
SRR515339	Arabidopsis thaliana	no
SRR515340	Arabidopsis thaliana	no
SRR515489	Arabidopsis thaliana	no
SRR515490	Arabidopsis thaliana	yes
SRR515491	Arabidopsis thaliana	yes
SRR515492	Arabidopsis thaliana	no
SRR515493	Arabidopsis thaliana	yes
SRR518039	Arabidopsis thaliana	yes
SRR518040	Arabidopsis thaliana	yes
SRR518041	Arabidopsis thaliana	yes
SRR518042	Arabidopsis thaliana	yes
SRR515074	Arabidopsis thaliana	yes
SRR515160	Arabidopsis thaliana	yes
SRR515175	Arabidopsis thaliana	yes
SRR515182	Arabidopsis thaliana	yes
SRR493097	Arabidopsis thaliana	no
SRR493098	Arabidopsis thaliana	no
SRR493101	Arabidopsis thaliana	yes
SRR493237	Arabidopsis thaliana	no
SRR493238	Arabidopsis thaliana	no
SRR493239	Arabidopsis thaliana	yes
SRR493240	Arabidopsis thaliana	yes
SRR493285	Arabidopsis thaliana	no
SRR444595	Arabidopsis thaliana	yes

SRA Run accession	Species	Is qualified?
SRR444596	Arabidopsis thaliana	yes
SRR444597	Arabidopsis thaliana	yes
SRR444598	Arabidopsis thaliana	yes
SRR444599	Arabidopsis thaliana	yes
SRR444600	Arabidopsis thaliana	yes
SRR444601	Arabidopsis thaliana	yes
SRR444602	Arabidopsis thaliana	yes
SRR445735	Arabidopsis thaliana	no
SRR445736	Arabidopsis thaliana	no
SRR445737	Arabidopsis thaliana	no
SRR445738	Arabidopsis thaliana	no
SRR446484	Arabidopsis thaliana	no
SRR446485	Arabidopsis thaliana	yes
SRR446486	Arabidopsis thaliana	yes
SRR446487	Arabidopsis thaliana	yes
SRR477075	Arabidopsis thaliana	yes
SRR477076	Arabidopsis thaliana	yes
SRR477077	Arabidopsis thaliana	yes
SRR477078	Arabidopsis thaliana	no
SRR477079	Arabidopsis thaliana	yes
SRR477080	Arabidopsis thaliana	no
SRR477081	Arabidopsis thaliana	no
SRR477082	Arabidopsis thaliana	no
SRR479032	Arabidopsis thaliana	yes
SRR479033	Arabidopsis thaliana	yes
SRR479034	Arabidopsis thaliana	yes
SRR479035	Arabidopsis thaliana	yes
SRR479036	Arabidopsis thaliana	yes
SRR479037	Arabidopsis thaliana	yes
SRR479038	Arabidopsis thaliana	yes
SRR493036	Arabidopsis thaliana	no
SRR493037	Arabidopsis thaliana	no
SRR493038	Arabidopsis thaliana	no
SRR493039	Arabidopsis thaliana	yes
SRR493040	Arabidopsis thaliana	yes
SRR493041	Arabidopsis thaliana	no
SRR493042	Arabidopsis thaliana	no
SRR493043	Arabidopsis thaliana	yes
SRR493044	Arabidopsis thaliana	no
SRR493045	Arabidopsis thaliana	yes
SRR493046	Arabidopsis thaliana	no
SRR493047	Arabidopsis thaliana	no
SRR501595	Arabidopsis thaliana	no
SRR501596	Arabidopsis thaliana	yes
SRR501594	Arabidopsis thaliana	yes

SRA Run accession	Species	Is qualified?
SRR501597	Arabidopsis thaliana	yes
SRR446027	Arabidopsis thaliana	yes
SRR446036	Arabidopsis thaliana	yes
SRR446037	Arabidopsis thaliana	yes
SRR446038	Arabidopsis thaliana	yes
SRR446039	Arabidopsis thaliana	no
SRR446040	Arabidopsis thaliana	yes
SRR446041	Arabidopsis thaliana	no
SRR446042	Arabidopsis thaliana	yes
SRR446043	Arabidopsis thaliana	no
SRR446044	Arabidopsis thaliana	yes
SRR446028	Arabidopsis thaliana	yes
SRR446029	Arabidopsis thaliana	yes
SRR446030	Arabidopsis thaliana	yes
SRR446031	Arabidopsis thaliana	yes
SRR446032	Arabidopsis thaliana	yes
SRR446033	Arabidopsis thaliana	no
SRR446034	Arabidopsis thaliana	yes
SRR446035	Arabidopsis thaliana	yes
SRR499985	Arabidopsis thaliana	no
SRR499983	Arabidopsis thaliana	no
SRR493286	Arabidopsis thaliana	yes
SRR493287	Arabidopsis thaliana	yes
SRR493288	Arabidopsis thaliana	yes
SRR493289	Arabidopsis thaliana	yes
SRR493290	Arabidopsis thaliana	yes
SRR499986	Arabidopsis thaliana	no
SRR499990	Arabidopsis thaliana	no
SRR499988	Arabidopsis thaliana	no
SRR499989	Arabidopsis thaliana	no
SRR499991	Arabidopsis thaliana	no
SRR499987	Arabidopsis thaliana	no
SRR392125	Arabidopsis thaliana	yes
SRR392126	Arabidopsis thaliana	yes
SRR392127	Arabidopsis thaliana	yes
SRR392128	Arabidopsis thaliana	yes
SRR392129	Arabidopsis thaliana	yes
SRR392130	Arabidopsis thaliana	yes
SRR392131	Arabidopsis thaliana	no
SRR392132	Arabidopsis thaliana	no
SRR392133	Arabidopsis thaliana	no
SRR394082	Arabidopsis thaliana	yes
SRR401413	Arabidopsis thaliana	no
SRR401414	Arabidopsis thaliana	no
SRR401415	Arabidopsis thaliana	no

SRA Run accession	Species	Is qualified?
SRR401416	Arabidopsis thaliana	no
SRR401417	Arabidopsis thaliana	no
SRR401418	Arabidopsis thaliana	no
SRR401419	Arabidopsis thaliana	no
SRR401420	Arabidopsis thaliana	no
SRR401421	Arabidopsis thaliana	no
SRR401422	Arabidopsis thaliana	no
SRR401423	Arabidopsis thaliana	no
SRR401424	Arabidopsis thaliana	no
SRR401425	Arabidopsis thaliana	no
SRR401426	Arabidopsis thaliana	no
SRR401427	Arabidopsis thaliana	no
SRR401428	Arabidopsis thaliana	no
SRR401429	Arabidopsis thaliana	no
SRR401430	Arabidopsis thaliana	no
SRR402994	Arabidopsis thaliana	no
SRR402995	Arabidopsis thaliana	no
SRR402996	Arabidopsis thaliana	no
SRR402997	Arabidopsis thaliana	no
SRR402998	Arabidopsis thaliana	no
SRR419182	Arabidopsis thaliana	no
SRR419183	Arabidopsis thaliana	no
SRR419184	Arabidopsis thaliana	no
SRR419185	Arabidopsis thaliana	no
SRR419186	Arabidopsis thaliana	no
SRR419187	Arabidopsis thaliana	no
SRR420813	Arabidopsis thaliana	no
SRR420814	Arabidopsis thaliana	no
SRR420815	Arabidopsis thaliana	no
SRR420816	Arabidopsis thaliana	no
SRR420817	Arabidopsis thaliana	no
SRR420818	Arabidopsis thaliana	no
SRR352212	Arabidopsis thaliana	no
SRR352213	Arabidopsis thaliana	no
SRR331219	Arabidopsis thaliana	no
SRR331224	Arabidopsis thaliana	yes
SRR314818	Arabidopsis thaliana	no
SRR332275	Arabidopsis thaliana	no
SRR332276	Arabidopsis thaliana	no
SRR332277	Arabidopsis thaliana	no
SRR332278	Arabidopsis thaliana	no
SRR332279	Arabidopsis thaliana	no
SRR332280	Arabidopsis thaliana	no
SRR345561	Arabidopsis thaliana	yes
SRR345562	Arabidopsis thaliana	yes

SRA Run accession	Species	Is qualified?
SRR345563	Arabidopsis thaliana	no
SRR345564	Arabidopsis thaliana	yes
SRR349697	Arabidopsis thaliana	yes
SRR349698	Arabidopsis thaliana	yes
SRR346552	Arabidopsis thaliana	yes
SRR346553	Arabidopsis thaliana	yes
SRR364390	Arabidopsis thaliana	no
SRR364389	Arabidopsis thaliana	no
SRR364391	Arabidopsis thaliana	no
SRR364392	Arabidopsis thaliana	no
SRR364393	Arabidopsis thaliana	no
SRR364394	Arabidopsis thaliana	no
SRR364395	Arabidopsis thaliana	no
SRR364396	Arabidopsis thaliana	no
SRR364397	Arabidopsis thaliana	no
SRR364398	Arabidopsis thaliana	no
SRR364399	Arabidopsis thaliana	no
SRR364400	Arabidopsis thaliana	no
SRR364401	Arabidopsis thaliana	no
SRR364402	Arabidopsis thaliana	no
SRR364403	Arabidopsis thaliana	no
SRR364404	Arabidopsis thaliana	no
SRR364405	Arabidopsis thaliana	no
SRR364406	Arabidopsis thaliana	no
SRR364407	Arabidopsis thaliana	no
SRR364408	Arabidopsis thaliana	no
SRR364409	Arabidopsis thaliana	no
SRR364465	Arabidopsis thaliana	no
SRR364466	Arabidopsis thaliana	no
SRR364467	Arabidopsis thaliana	no
SRR364468	Arabidopsis thaliana	no
SRR364469	Arabidopsis thaliana	no
SRR364470	Arabidopsis thaliana	no
SRR388665	Arabidopsis thaliana	yes
SRR388666	Arabidopsis thaliana	yes
SRR388667	Arabidopsis thaliana	no
SRR388668	Arabidopsis thaliana	yes
SRR388669	Arabidopsis thaliana	yes
SRR388670	Arabidopsis thaliana	yes
SRR390302	Arabidopsis thaliana	no
SRR390303	Arabidopsis thaliana	no
SRR390304	Arabidopsis thaliana	no
SRR390306	Arabidopsis thaliana	no
SRR390307	Arabidopsis thaliana	no
SRR390308	Arabidopsis thaliana	no

SRA Run accession	Species	Is qualified?
SRR390309	Arabidopsis thaliana	no
SRR390311	Arabidopsis thaliana	no
SRR390312	Arabidopsis thaliana	no
SRR390313	Arabidopsis thaliana	no
SRR392118	Arabidopsis thaliana	yes
SRR392119	Arabidopsis thaliana	yes
SRR392120	Arabidopsis thaliana	yes
SRR392121	Arabidopsis thaliana	yes
SRR392122	Arabidopsis thaliana	yes
SRR392123	Arabidopsis thaliana	yes
SRR392124	Arabidopsis thaliana	yes
SRR331227	Arabidopsis thaliana	yes
SRR364677	Arabidopsis thaliana	no
SRR331228	Arabidopsis thaliana	yes
SRR331229	Arabidopsis thaliana	yes
SRR352214	Arabidopsis thaliana	no
SRR352215	Arabidopsis thaliana	no
SRR364678	Arabidopsis thaliana	no
SRR100209	Arabidopsis thaliana	yes
SRR100211	Arabidopsis thaliana	yes
SRR100210	Arabidopsis thaliana	yes
SRR100212	Arabidopsis thaliana	no
SRR100213	Arabidopsis thaliana	yes
SRR100214	Arabidopsis thaliana	yes
SRR100215	Arabidopsis thaliana	yes
SRR100216	Arabidopsis thaliana	yes
SRR189800	Arabidopsis thaliana	no
SRR189801	Arabidopsis thaliana	no
SRR189802	Arabidopsis thaliana	no
SRR189803	Arabidopsis thaliana	no
SRR307067	Arabidopsis thaliana	yes
SRR307068	Arabidopsis thaliana	yes
SRR307070	Arabidopsis thaliana	yes
SRR307069	Arabidopsis thaliana	yes
SRR307071	Arabidopsis thaliana	yes
SRR307072	Arabidopsis thaliana	yes
SRR307073	Arabidopsis thaliana	yes
SRR307074	Arabidopsis thaliana	yes
SRR307075	Arabidopsis thaliana	yes
SRR307076	Arabidopsis thaliana	yes
SRR309145	Arabidopsis thaliana	no
SRR309146	Arabidopsis thaliana	no
SRR309147	Arabidopsis thaliana	no
SRR309148	Arabidopsis thaliana	no
SRR309149	Arabidopsis thaliana	no

SRA Run accession	Species	Is qualified?
SRR309150	Arabidopsis thaliana	no
SRR309151	Arabidopsis thaliana	no
SRR309152	Arabidopsis thaliana	no
SRR309153	Arabidopsis thaliana	no
SRR309154	Arabidopsis thaliana	no
SRR309155	Arabidopsis thaliana	no
SRR309156	Arabidopsis thaliana	no
SRR309157	Arabidopsis thaliana	no
SRR309158	Arabidopsis thaliana	no
SRR309159	Arabidopsis thaliana	no
SRR309160	Arabidopsis thaliana	no
SRR309161	Arabidopsis thaliana	no
SRR309162	Arabidopsis thaliana	no
SRR309163	Arabidopsis thaliana	no
SRR309164	Arabidopsis thaliana	no
SRR309165	Arabidopsis thaliana	no
SRR309166	Arabidopsis thaliana	no
SRR309167	Arabidopsis thaliana	no
SRR309168	Arabidopsis thaliana	no
SRR309169	Arabidopsis thaliana	no
SRR309170	Arabidopsis thaliana	no
SRR309171	Arabidopsis thaliana	no
SRR309172	Arabidopsis thaliana	no
SRR309173	Arabidopsis thaliana	no
SRR309174	Arabidopsis thaliana	no
SRR309175	Arabidopsis thaliana	no
SRR309176	Arabidopsis thaliana	no
SRR309177	Arabidopsis thaliana	no
SRR309178	Arabidopsis thaliana	no
SRR309179	Arabidopsis thaliana	no
SRR309180	Arabidopsis thaliana	no
SRR309181	Arabidopsis thaliana	no
SRR309182	Arabidopsis thaliana	no
SRR309183	Arabidopsis thaliana	no
SRR309184	Arabidopsis thaliana	no
SRR309185	Arabidopsis thaliana	no
SRR309186	Arabidopsis thaliana	no
SRR314813	Arabidopsis thaliana	yes
SRR314814	Arabidopsis thaliana	yes
SRR314815	Arabidopsis thaliana	yes
SRR314816	Arabidopsis thaliana	yes
SRR314817	Arabidopsis thaliana	no
SRR051926	Arabidopsis thaliana	no
SRR051927	Arabidopsis thaliana	no
SRR068956	Arabidopsis thaliana	no

SRA Run accession	Species	Is qualified?
SRR068957	Arabidopsis thaliana	no
SRR068958	Arabidopsis thaliana	no
SRR068959	Arabidopsis thaliana	no
SRR068960	Arabidopsis thaliana	no
SRR068961	Arabidopsis thaliana	no
SRR068962	Arabidopsis thaliana	no
SRR068963	Arabidopsis thaliana	no
SRR068964	Arabidopsis thaliana	no
SRR068965	Arabidopsis thaliana	yes
SRR068966	Arabidopsis thaliana	yes
SRR068967	Arabidopsis thaliana	yes
SRR068968	Arabidopsis thaliana	no
SRR068969	Arabidopsis thaliana	yes
SRR068970	Arabidopsis thaliana	yes
SRR068971	Arabidopsis thaliana	no
SRR068972	Arabidopsis thaliana	no
SRR068973	Arabidopsis thaliana	no
SRR068974	Arabidopsis thaliana	no
SRR068975	Arabidopsis thaliana	no
SRR068976	Arabidopsis thaliana	no
SRR068977	Arabidopsis thaliana	no
SRR068978	Arabidopsis thaliana	no
SRR068979	Arabidopsis thaliana	no
SRR068980	Arabidopsis thaliana	no
SRR068981	Arabidopsis thaliana	no
SRR068982	Arabidopsis thaliana	no
SRR068983	Arabidopsis thaliana	no
SRR068984	Arabidopsis thaliana	no
SRR068985	Arabidopsis thaliana	no
SRR068986	Arabidopsis thaliana	no
SRR068987	Arabidopsis thaliana	no
SRR068988	Arabidopsis thaliana	no
SRR068989	Arabidopsis thaliana	no
SRR068990	Arabidopsis thaliana	no
SRR519449	Vitis vinifera	yes
SRR519450	Vitis vinifera	yes
SRR519451	Vitis vinifera	yes
SRR519452	Vitis vinifera	yes
SRR520376	Vitis vinifera	yes
SRR519453	Vitis vinifera	yes
SRR519454	Vitis vinifera	yes
SRR519455	Vitis vinifera	yes
SRR519456	Vitis vinifera	yes
SRR520374	Vitis vinifera	yes
SRR493740	Vitis vinifera	no

SRA Run accession	Species	Is qualified?
SRR493741	Vitis vinifera	yes
SRR493742	Vitis vinifera	yes
SRR493743	Vitis vinifera	yes
SRR493744	Vitis vinifera	yes
SRR493745	Vitis vinifera	yes
SRR493746	Vitis vinifera	yes
SRR522298	Vitis vinifera	yes
SRR522471	Vitis vinifera	yes
SRR522472	Vitis vinifera	yes
SRR522473	Vitis vinifera	yes
SRR522474	Vitis vinifera	yes
SRR522475	Vitis vinifera	yes
SRR522477	Vitis vinifera	yes
SRR522478	Vitis vinifera	yes
SRR522479	Vitis vinifera	yes
SRR522484	Vitis vinifera	yes
SRR520378	Vitis vinifera	yes
SRR520379	Vitis vinifera	yes
SRR520380	Vitis vinifera	yes
SRR520381	Vitis vinifera	yes
SRR520382	Vitis vinifera	yes
SRR520384	Vitis vinifera	yes
SRR520385	Vitis vinifera	yes
SRR520386	Vitis vinifera	yes
SRR520387	Vitis vinifera	yes
SRR520388	Vitis vinifera	yes
SRR653087	Vitis vinifera	no
SRR653088	Vitis vinifera	no
SRR653089	Vitis vinifera	no
SRR653090	Vitis vinifera	no
SRR088877	Medicago truncatula	no
SRR088878	Medicago truncatula	yes
SRR391514	Medicago truncatula	yes
SRR391515	Medicago truncatula	yes
SRR391516	Medicago truncatula	yes
SRR391517	Medicago truncatula	yes
SRR391518	Medicago truncatula	yes
SRR391519	Medicago truncatula	yes
SRR391520	Medicago truncatula	yes
SRR391521	Medicago truncatula	yes
SRR504352	Medicago truncatula	yes
SRR504353	Medicago truncatula	yes
SRR504354	Medicago truncatula	yes
SRR504355	Medicago truncatula	yes
SRR504356	Medicago truncatula	yes

SRA Run accession	Species	Is qualified?
SRR504357	Medicago truncatula	yes
SRR670345	Medicago truncatula	yes
SRR670346	Medicago truncatula	yes
SRR670347	Medicago truncatula	yes
SRR670348	Medicago truncatula	yes
SRR670349	Medicago truncatula	yes
SRR670350	Medicago truncatula	yes
SRR670351	Medicago truncatula	yes
SRR670352	Medicago truncatula	yes
SRR670353	Medicago truncatula	yes
SRR670354	Medicago truncatula	yes
SRR670355	Medicago truncatula	yes
SRR670356	Medicago truncatula	yes
SRR670357	Medicago truncatula	yes
SRR670358	Medicago truncatula	yes
SRR670383	Medicago truncatula	yes
SRR670400	Medicago truncatula	yes
SRR670403	Medicago truncatula	yes
SRR670404	Medicago truncatula	yes
DRR001963	Oryza sativa	yes
DRR001964	Oryza sativa	yes
DRR001965	Oryza sativa	yes
SRR576931	Oryza sativa	no
SRR576932	Oryza sativa	no
SRR611648	Oryza sativa	no
SRR611649	Oryza sativa	no
SRR611650	Oryza sativa	no
SRR611651	Oryza sativa	no
SRR711312	Oryza sativa	no
SRR711313	Oryza sativa	no
SRR711314	Oryza sativa	no
SRR711315	Oryza sativa	no
SRR711316	Oryza sativa	no
SRR711317	Oryza sativa	no
SRR711318	Oryza sativa	no
SRR711319	Oryza sativa	no
SRR711320	Oryza sativa	no
SRR711321	Oryza sativa	no
SRR711322	Oryza sativa	yes
SRR711323	Oryza sativa	no
SRR711324	Oryza sativa	no
SRR711325	Oryza sativa	no
SRR711326	Oryza sativa	yes
SRR711327	Oryza sativa	yes
SRR711328	Oryza sativa	yes

SRA Run accession	Species	Is qualified?
SRR711329	Oryza sativa	no
SRR711330	Oryza sativa	no
SRR037712	Oryza sativa	no
SRR037713	Oryza sativa	no
SRR037714	Oryza sativa	no
SRR037715	Oryza sativa	no
SRR037716	Oryza sativa	no
SRR037717	Oryza sativa	no
SRR037718	Oryza sativa	no
SRR037719	Oryza sativa	no
SRR037720	Oryza sativa	no
SRR037721	Oryza sativa	no
SRR037722	Oryza sativa	no
SRR037723	Oryza sativa	no
SRR037724	Oryza sativa	no
SRR037726	Oryza sativa	no
SRR037727	Oryza sativa	no
SRR037728	Oryza sativa	no
SRR037729	Oryza sativa	no
SRR037730	Oryza sativa	no
SRR037731	Oryza sativa	no
SRR037732	Oryza sativa	no
SRR037733	Oryza sativa	no
SRR037734	Oryza sativa	no
SRR037735	Oryza sativa	no
SRR037736	Oryza sativa	no
SRR037737	Oryza sativa	no
SRR037738	Oryza sativa	no
SRR037739	Oryza sativa	no
SRR447117	Oryza sativa	no
SRR447118	Oryza sativa	no
SRR447119	Oryza sativa	no
SRR447120	Oryza sativa	no
SRR447121	Oryza sativa	no
SRR447122	Oryza sativa	no
SRR447123	Oryza sativa	no
SRR447124	Oryza sativa	no
SRR447125	Oryza sativa	no
SRR447126	Oryza sativa	no
SRR504364	Oryza sativa	yes
SRR504365	Oryza sativa	no
SRR504366	Oryza sativa	yes
SRR504367	Oryza sativa	yes
SRR504368	Oryza sativa	yes
SRR504369	Oryza sativa	yes

SRA Run accession	Species	Is qualified?
SRR504370	Oryza sativa	yes
SRR504371	Oryza sativa	no
ERR194168	Oryza sativa	no
SRR306316	Oryza sativa	no
SRR306317	Oryza sativa	no
SRR306318	Oryza sativa	no
SRR306319	Oryza sativa	no
SRR306320	Oryza sativa	no
SRR306321	Oryza sativa	no
SRR306322	Oryza sativa	no
SRR306323	Oryza sativa	no
SRR306324	Oryza sativa	no
SRR306325	Oryza sativa	no
SRR358791	Oryza sativa	no
SRR358792	Oryza sativa	no
SRR358793	Oryza sativa	no
SRR358794	Oryza sativa	no
SRR358795	Oryza sativa	yes
SRR358796	Oryza sativa	yes
SRR358797	Oryza sativa	yes
SRR358798	Oryza sativa	yes
SRR408729	Oryza sativa	yes
SRR408730	Oryza sativa	no
SRR408731	Oryza sativa	no
SRR408732	Oryza sativa	no
SRR408733	Oryza sativa	no
SRR408734	Oryza sativa	no
SRR408735	Oryza sativa	no
SRR408736	Oryza sativa	no
SRR408737	Oryza sativa	no
SRR408738	Oryza sativa	no
SRR408739	Oryza sativa	no
SRR408740	Oryza sativa	no
SRR408741	Oryza sativa	no
SRR408742	Oryza sativa	no
SRR408743	Oryza sativa	no
SRR408744	Oryza sativa	no
SRR408745	Oryza sativa	no
SRR408746	Oryza sativa	no
SRR408747	Oryza sativa	no
SRR408748	Oryza sativa	yes
SRR408749	Oryza sativa	no
SRR352184	Oryza sativa	yes
SRR352187	Oryza sativa	yes
SRR352189	Oryza sativa	no

SRA Run accession	Species	Is qualified?
SRR352190	Oryza sativa	yes
SRR352192	Oryza sativa	no
SRR352194	Oryza sativa	no
SRR352204	Oryza sativa	yes
SRR352206	Oryza sativa	yes
SRR352207	Oryza sativa	yes
SRR352209	Oryza sativa	yes
SRR352211	Oryza sativa	no
SRR305463	Oryza sativa	no
SRR305476	Oryza sativa	no
SRR305477	Oryza sativa	no
SRR305891	Oryza sativa	no
SRR305892	Oryza sativa	no
SRR305893	Oryza sativa	no
DRR000753	Oryza sativa	yes
DRR000754	Oryza sativa	yes
DRR000755	Oryza sativa	yes
DRR000758	Oryza sativa	yes
DRR000745	Oryza sativa	yes
DRR000746	Oryza sativa	yes
DRR000747	Oryza sativa	yes
DRR000748	Oryza sativa	yes
DRR000749	Oryza sativa	yes
DRR000761	Oryza sativa	yes
DRR000762	Oryza sativa	yes
DRR000763	Oryza sativa	yes
DRR000764	Oryza sativa	yes
DRR000765	Oryza sativa	yes
DRR000766	Oryza sativa	yes
DRR000769	Oryza sativa	yes
DRR000767	Oryza sativa	yes
DRR000768	Oryza sativa	yes
DRR000750	Oryza sativa	yes
DRR000751	Oryza sativa	yes
DRR000752	Oryza sativa	yes
DRR000759	Oryza sativa	yes
DRR000760	Oryza sativa	yes
DRR000756	Oryza sativa	yes
DRR000757	Oryza sativa	yes
DRR001024	Oryza sativa	no
DRR001025	Oryza sativa	no
DRR001026	Oryza sativa	yes
DRR001027	Oryza sativa	no
DRR001028	Oryza sativa	yes
DRR001029	Oryza sativa	no

SRA Run accession	Species	Is qualified?
DRR001030	Oryza sativa	no
DRR001031	Oryza sativa	yes
DRR001032	Oryza sativa	no
DRR001033	Oryza sativa	yes
DRR001034	Oryza sativa	yes
DRR001035	Oryza sativa	yes
DRR001036	Oryza sativa	no
DRR001037	Oryza sativa	yes
DRR001038	Oryza sativa	yes
DRR001039	Oryza sativa	no
DRR001040	Oryza sativa	yes
DRR001041	Oryza sativa	yes
DRR001042	Oryza sativa	yes
DRR001043	Oryza sativa	no
DRR001044	Oryza sativa	yes
DRR001045	Oryza sativa	no
DRR001046	Oryza sativa	no
DRR001047	Oryza sativa	no
DRR001048	Oryza sativa	no
DRR001049	Oryza sativa	no
DRR001050	Oryza sativa	no
DRR001051	Oryza sativa	no
SRR037711	Oryza sativa	yes
SRR037725	Oryza sativa	yes
SRR037740	Oryza sativa	yes
SRR037741	Oryza sativa	yes
SRR037742	Oryza sativa	yes
SRR037743	Oryza sativa	yes
SRR037744	Oryza sativa	no
SRR037745	Oryza sativa	no
SRR032097	Oryza sativa	no
SRR032098	Oryza sativa	no
SRR034580	Oryza sativa	yes
SRR034581	Oryza sativa	yes
SRR034582	Oryza sativa	yes
SRR034583	Oryza sativa	yes
SRR034584	Oryza sativa	no
SRR034585	Oryza sativa	no
SRR034586	Oryza sativa	no
SRR034587	Oryza sativa	no
SRR034588	Oryza sativa	no
SRR034589	Oryza sativa	no
SRR034590	Oryza sativa	no
SRR034591	Oryza sativa	no
SRR034592	Oryza sativa	no

SRA Run accession	Species	Is qualified?
SRR034593	Oryza sativa	no
SRR034594	Oryza sativa	no
SRR034595	Oryza sativa	no
SRR034596	Oryza sativa	no
SRR034597	Oryza sativa	no
SRR034598	Oryza sativa	no
SRR034599	Oryza sativa	no
SRR042529	Oryza sativa	no
SRR037232	Oryza sativa	no
SRR037233	Oryza sativa	no
SRR037234	Oryza sativa	no
SRR037235	Oryza sativa	no
SRR037236	Oryza sativa	no
SRR037237	Oryza sativa	no
SRR037238	Oryza sativa	no
SRR122108	Solanum tuberosum	yes
SRR122109	Solanum tuberosum	no
SRR122110	Solanum tuberosum	yes
SRR122111	Solanum tuberosum	yes
SRR122112	Solanum tuberosum	yes
SRR122113	Solanum tuberosum	no
SRR122114	Solanum tuberosum	yes
SRR122115	Solanum tuberosum	yes
SRR122116	Solanum tuberosum	yes
SRR122117	Solanum tuberosum	no
SRR122118	Solanum tuberosum	no
SRR122119	Solanum tuberosum	yes
SRR122120	Solanum tuberosum	yes
SRR122121	Solanum tuberosum	no
SRR122122	Solanum tuberosum	yes
SRR122123	Solanum tuberosum	no
SRR122124	Solanum tuberosum	no
SRR122125	Solanum tuberosum	yes
SRR122126	Solanum tuberosum	yes
SRR122127	Solanum tuberosum	no
SRR122128	Solanum tuberosum	no
SRR122129	Solanum tuberosum	no
SRR122130	Solanum tuberosum	yes
SRR122131	Solanum tuberosum	yes
SRR122132	Solanum tuberosum	yes
SRR122133	Solanum tuberosum	no
SRR122134	Solanum tuberosum	yes
SRR122135	Solanum tuberosum	yes
SRR122136	Solanum tuberosum	yes
SRR122137	Solanum tuberosum	yes

SRA Run accession	Species	Is qualified?
SRR122138	Solanum tuberosum	no
SRR122139	Solanum tuberosum	yes
SRR122140	Solanum tuberosum	yes
SRR124121	Solanum tuberosum	no
SRR124126	Solanum tuberosum	no
SRR124127	Solanum tuberosum	yes
SRR124130	Solanum tuberosum	yes
SRR124131	Solanum tuberosum	yes
SRR124132	Solanum tuberosum	yes
SRR124138	Solanum tuberosum	yes
SRR184099	Solanum tuberosum	yes
SRR184100	Solanum tuberosum	yes
SRR184101	Solanum tuberosum	no
SRR184102	Solanum tuberosum	no
SRR184103	Solanum tuberosum	no
SRR184104	Solanum tuberosum	no
SRR329935	Solanum tuberosum	no
SRR331231	Solanum tuberosum	no
SRR331743	Solanum tuberosum	no
SRR331744	Solanum tuberosum	no
SRR331745	Solanum tuberosum	no
SRR331746	Solanum tuberosum	no
SRR331824	Solanum tuberosum	no
SRR331863	Solanum tuberosum	no
SRR331947	Solanum tuberosum	no
SRR335407	Solanum tuberosum	no
SRR825895	Solanum tuberosum	no
SRR825896	Solanum tuberosum	no
SRR825897	Solanum tuberosum	no
SRR825898	Solanum tuberosum	no
SRR825899	Solanum tuberosum	no
SRR825900	Solanum tuberosum	no
SRR825901	Solanum tuberosum	no
SRR825902	Solanum tuberosum	no
SRR825903	Solanum tuberosum	no
SRR825904	Solanum tuberosum	no
SRR825905	Solanum tuberosum	no
SRR825906	Solanum tuberosum	no
SRR825907	Solanum tuberosum	no
SRR825908	Solanum tuberosum	no
SRR825909	Solanum tuberosum	no
SRR825910	Solanum tuberosum	no
SRR825911	Solanum tuberosum	no
SRR825912	Solanum tuberosum	no
SRR825913	Solanum tuberosum	no

SRA Run accession	Species	Is qualified?
SRR825914	Solanum tuberosum	no
SRR825915	Solanum tuberosum	no
SRR825916	Solanum tuberosum	no
SRR863371	Solanum tuberosum	no
SRR863697	Solanum tuberosum	no
SRR863698	Solanum tuberosum	yes
SRR864315	Solanum tuberosum	yes
SRR864485	Solanum tuberosum	no
SRR864713	Solanum tuberosum	yes
SRR865071	Solanum tuberosum	yes
SRR865299	Solanum tuberosum	yes
SRR865383	Solanum tuberosum	no
SRR865536	Solanum tuberosum	yes
SRR865575	Solanum tuberosum	no
SRR865691	Solanum tuberosum	no
SRR865788	Solanum tuberosum	no
SRR865843	Solanum tuberosum	no
SRR865902	Solanum tuberosum	no
SRR865992	Solanum tuberosum	no
SRR866043	Solanum tuberosum	no
SRR866220	Solanum tuberosum	no
SRR866226	Solanum tuberosum	no
SRR866232	Solanum tuberosum	no
SRR866237	Solanum tuberosum	no
SRR866242	Solanum tuberosum	no
SRR866243	Solanum tuberosum	no
SRR866244	Solanum tuberosum	no
SRR866245	Solanum tuberosum	no
SRR866250	Solanum tuberosum	no
SRR866252	Solanum tuberosum	no
SRR866253	Solanum tuberosum	no
SRR866254	Solanum tuberosum	no
SRR866256	Solanum tuberosum	no
SRR866257	Solanum tuberosum	no
SRR866258	Solanum tuberosum	no
SRR866259	Solanum tuberosum	no
SRR866266	Solanum tuberosum	no
SRR866268	Solanum tuberosum	no
SRR866275	Solanum tuberosum	no
DRR001053	Sorghum bicolor	yes
DRR001054	Sorghum bicolor	yes
DRR001055	Sorghum bicolor	yes
SRR299227	Sorghum bicolor	yes
SRR299228	Sorghum bicolor	yes
SRR299229	Sorghum bicolor	yes

SRA Run accession	Species	Is qualified?
SRR299230	Sorghum bicolor	yes
SRR299231	Sorghum bicolor	yes
SRR299232	Sorghum bicolor	yes
SRR299233	Sorghum bicolor	yes
SRR299234	Sorghum bicolor	yes
SRR299235	Sorghum bicolor	yes
SRR299236	Sorghum bicolor	yes
SRR299237	Sorghum bicolor	yes
SRR299238	Sorghum bicolor	yes
SRR299239	Sorghum bicolor	yes
SRR299240	Sorghum bicolor	yes
SRR299241	Sorghum bicolor	yes
SRR299242	Sorghum bicolor	yes
SRR299243	Sorghum bicolor	yes
SRR299244	Sorghum bicolor	yes
SRR299245	Sorghum bicolor	yes
SRR299246	Sorghum bicolor	yes
SRR299247	Sorghum bicolor	yes
SRR299248	Sorghum bicolor	yes
SRR299249	Sorghum bicolor	yes
SRR299250	Sorghum bicolor	yes
SRR349643	Sorghum bicolor	yes
SRR349644	Sorghum bicolor	yes
SRR349645	Sorghum bicolor	yes
SRR349646	Sorghum bicolor	yes
SRR349754	Sorghum bicolor	yes
SRR349767	Sorghum bicolor	yes
SRR349768	Sorghum bicolor	yes
SRR349769	Sorghum bicolor	yes
SRR349771	Sorghum bicolor	yes
SRR349772	Sorghum bicolor	yes
SRR038864	Glycine max	yes
SRR038865	Glycine max	yes
SRR038866	Glycine max	yes
SRR038867	Glycine max	yes
SRR043416	Glycine max	no
SRR043417	Glycine max	no
SRR043418	Glycine max	no
SRR043419	Glycine max	no
SRR1174205	Glycine max	no
SRR1174207	Glycine max	yes
SRR1174208	Glycine max	yes
SRR1174209	Glycine max	yes
SRR1174211	Glycine max	yes
SRR1174212	Glycine max	yes

SRA Run accession	Species	Is qualified?
SRR1174213	Glycine max	no
SRR1174214	Glycine max	yes
SRR1174215	Glycine max	no
SRR1174216	Glycine max	no
SRR1174217	Glycine max	no
SRR1174219	Glycine max	no
SRR1174220	Glycine max	no
SRR1174233	Glycine max	yes
SRR203030	Glycine max	yes
SRR203031	Glycine max	yes
SRR203032	Glycine max	yes
SRR203033	Glycine max	yes
SRR203034	Glycine max	yes
SRR203035	Glycine max	yes
SRR203036	Glycine max	yes
SRR203037	Glycine max	yes
SRR203038	Glycine max	yes
SRR203337	Glycine max	no
SRR203338	Glycine max	no
SRR203339	Glycine max	no
SRR203340	Glycine max	no
SRR203341	Glycine max	no
SRR203342	Glycine max	no
SRR203343	Glycine max	no
SRR203344	Glycine max	no
SRR203345	Glycine max	no
SRR203346	Glycine max	no
SRR203351	Glycine max	yes
SRR203352	Glycine max	yes
SRR203353	Glycine max	yes
SRR203354	Glycine max	yes
SRR203355	Glycine max	yes
SRR203356	Glycine max	yes
SRR203357	Glycine max	yes
SRR203358	Glycine max	yes
SRR203359	Glycine max	yes
SRR203360	Glycine max	yes
SRR203361	Glycine max	yes
SRR203362	Glycine max	yes
SRR203363	Glycine max	no
SRR203364	Glycine max	no
SRR203365	Glycine max	no
SRR203366	Glycine max	no
SRR203367	Glycine max	no
SRR203368	Glycine max	no

SRA Run accession	Species	Is qualified?
SRR203369	Glycine max	no
SRR243572	Glycine max	no
SRR243573	Glycine max	no
SRR243574	Glycine max	no
SRR324698	Glycine max	no
SRR324699	Glycine max	no
SRR324700	Glycine max	no
SRR324701	Glycine max	no
SRR352327	Glycine max	no
SRR352328	Glycine max	no
SRR357245	Glycine max	no
SRR357246	Glycine max	no
SRR361417	Glycine max	no
SRR389188	Glycine max	yes
SRR389189	Glycine max	no
SRR389190	Glycine max	no
SRR389191	Glycine max	no
SRR389192	Glycine max	no
SRR391535	Glycine max	no
SRR391536	Glycine max	no
SRR391537	Glycine max	no
SRR391538	Glycine max	no
SRR391539	Glycine max	no
SRR391541	Glycine max	no
SRR394316	Glycine max	no
SRR394317	Glycine max	no
SRR394318	Glycine max	no
SRR394319	Glycine max	yes
SRR445528	Glycine max	no
SRR445529	Glycine max	no
SRR446583	Glycine max	no
SRR446584	Glycine max	no
SRR447748	Glycine max	no
SRR447749	Glycine max	no
SRR605683	Glycine max	no
SRR605684	Glycine max	no
SRR605685	Glycine max	no
SRR605686	Glycine max	no
SRR605687	Glycine max	no
SRR605688	Glycine max	no
SRR605689	Glycine max	no
SRR605690	Glycine max	no
SRR605691	Glycine max	no
SRR605692	Glycine max	no
SRR605693	Glycine max	no

SRA Run accession	Species	Is qualified?
SRR605694	Glycine max	no
SRR610280	Glycine max	no
SRR610284	Glycine max	no
SRR639162	Glycine max	yes
SRR639163	Glycine max	yes
SRR639164	Glycine max	yes
SRR639165	Glycine max	yes
SRR639166	Glycine max	yes
SRR639167	Glycine max	yes
SRR639168	Glycine max	yes
SRR639169	Glycine max	yes
SRR639170	Glycine max	yes
SRR639171	Glycine max	yes
SRR639172	Glycine max	yes
SRR639173	Glycine max	yes
SRR639174	Glycine max	yes
SRR639175	Glycine max	yes
SRR639176	Glycine max	yes
SRR639177	Glycine max	yes
SRR646494	Glycine max	no
SRR646495	Glycine max	no
SRR646496	Glycine max	no
SRR646497	Glycine max	no
SRR646498	Glycine max	no
SRR646499	Glycine max	no
SRR646500	Glycine max	no
SRR646501	Glycine max	no
SRR646502	Glycine max	no
SRR646503	Glycine max	no
SRR646504	Glycine max	no
SRR646505	Glycine max	no
SRR646506	Glycine max	no
SRR646507	Glycine max	no
SRR646508	Glycine max	no
SRR646509	Glycine max	no
SRR646510	Glycine max	no
SRR648493	Glycine max	no
SRR648494	Glycine max	no
SRR648495	Glycine max	no
SRR648496	Glycine max	no
SRR648497	Glycine max	no
SRR648498	Glycine max	no
SRR648499	Glycine max	no
SRR648500	Glycine max	no
SRR648501	Glycine max	no

SRA Run accession	Species	Is qualified?
SRR648502	Glycine max	no
SRR648503	Glycine max	no
SRR648504	Glycine max	no
SRR648505	Glycine max	no
SRR648506	Glycine max	no
SRR648507	Glycine max	no
SRR648508	Glycine max	no
SRR648509	Glycine max	no
SRR648510	Glycine max	no
SRR648511	Glycine max	no
SRR648512	Glycine max	no
SRR648513	Glycine max	no
SRR648514	Glycine max	no
SRR648515	Glycine max	no
SRR648516	Glycine max	no
SRR824155	Glycine max	no
SRR824156	Glycine max	no
SRR824157	Glycine max	no
SRR824158	Glycine max	no
SRR824159	Glycine max	no
SRR824160	Glycine max	no
SRR824161	Glycine max	no
SRR824162	Glycine max	no
SRR824163	Glycine max	no
SRR827653	Glycine max	yes
SRR827654	Glycine max	yes
SRR827655	Glycine max	yes
SRR827656	Glycine max	yes
SRR827657	Glycine max	yes
SRR827658	Glycine max	yes
SRR827659	Glycine max	yes
SRR827660	Glycine max	yes
SRR827661	Glycine max	yes
SRR827662	Glycine max	yes
SRR827663	Glycine max	yes
SRR827664	Glycine max	yes
SRR827665	Glycine max	yes
SRR827666	Glycine max	yes
SRR827667	Glycine max	yes
SRR827668	Glycine max	yes
SRR827669	Glycine max	yes
SRR827670	Glycine max	yes
SRR827671	Glycine max	yes
SRR827672	Glycine max	no
SRR827673	Glycine max	yes

SRA Run accession	Species	Is qualified?
SRR827674	Glycine max	yes
SRR827675	Glycine max	yes
SRR827676	Glycine max	yes
SRR827677	Glycine max	yes
SRR827678	Glycine max	yes
SRR827679	Glycine max	yes
SRR827680	Glycine max	yes
SRR827681	Glycine max	yes
SRR827682	Glycine max	yes
SRR827683	Glycine max	yes
SRR827684	Glycine max	yes
SRR827685	Glycine max	yes
SRR827686	Glycine max	yes
SRR827687	Glycine max	yes
SRR827688	Glycine max	yes
SRR827689	Glycine max	yes
SRR827690	Glycine max	yes
SRR827691	Glycine max	yes
SRR827692	Glycine max	yes
SRR827693	Glycine max	yes
SRR827694	Glycine max	yes
SRR827695	Glycine max	yes
SRR827696	Glycine max	yes
SRR827697	Glycine max	yes
SRR827698	Glycine max	yes
SRR830182	Glycine max	yes
SRR830183	Glycine max	yes
SRR830184	Glycine max	yes
SRR830185	Glycine max	yes
SRR830186	Glycine max	yes
SRR830187	Glycine max	yes
SRR830188	Glycine max	yes
SRR830189	Glycine max	yes
SRR830190	Glycine max	yes
SRR830191	Glycine max	no
SRR830192	Glycine max	no
SRR830193	Glycine max	no
SRR830194	Glycine max	yes
SRR830195	Glycine max	yes
SRR830196	Glycine max	yes
SRR830197	Glycine max	yes
SRR830198	Glycine max	yes
SRR830199	Glycine max	yes
SRR830200	Glycine max	yes
SRR830201	Glycine max	yes

SRA Run accession	Species	Is qualified?
SRR830202	Glycine max	yes
SRR830203	Glycine max	yes
SRR830204	Glycine max	yes
SRR830205	Glycine max	yes
SRR830206	Glycine max	yes
SRR830207	Glycine max	yes
SRR830208	Glycine max	yes
SRR830209	Glycine max	yes
SRR830210	Glycine max	yes
SRR830211	Glycine max	yes
SRR847313	Glycine max	no
SRR848921	Glycine max	no
SRR848922	Glycine max	no
SRR849498	Glycine max	no
SRR849499	Glycine max	no
SRR863029	Glycine max	no
SRR863032	Glycine max	no
SRR887342	Glycine max	yes
SRR891238	Glycine max	yes
SRR891247	Glycine max	yes
SRR891260	Glycine max	yes
SRR891284	Glycine max	yes
SRR891292	Glycine max	yes
SRR923882	Glycine max	yes
SRR923884	Glycine max	yes
SRR923885	Glycine max	yes
SRR923886	Glycine max	yes
SRR923887	Glycine max	yes
SRR923889	Glycine max	yes
SRR923890	Glycine max	yes
SRR923891	Glycine max	yes
SRR923892	Glycine max	yes
SRR923893	Glycine max	yes
SRR923894	Glycine max	yes
SRR923896	Glycine max	yes
SRR923897	Glycine max	yes
SRR923898	Glycine max	yes
SRR923899	Glycine max	yes
SRR923900	Glycine max	yes
SRR923901	Glycine max	yes
SRR923903	Glycine max	yes
SRR923904	Glycine max	yes
SRR924099	Glycine max	no
SRR924144	Glycine max	no
SRR925830	Glycine max	no

SRA Run accession	Species	Is qualified?
SRR925831	Glycine max	no
SRR925832	Glycine max	no
SRR925833	Glycine max	no
SRR925834	Glycine max	no
SRR925835	Glycine max	no
SRR925836	Glycine max	no
SRR925837	Glycine max	no
SRR925838	Glycine max	no
SRR925839	Glycine max	no
SRR925840	Glycine max	no
SRR925841	Glycine max	no
SRR925842	Glycine max	no
SRR925843	Glycine max	no
SRR925844	Glycine max	no
SRR925845	Glycine max	no
SRR925846	Glycine max	no
SRR925847	Glycine max	no
SRR925848	Glycine max	no
SRR925849	Glycine max	no
SRR925850	Glycine max	no
SRR925851	Glycine max	no
SRR926169	Glycine max	no
SRR926170	Glycine max	no
SRR926171	Glycine max	no
SRR926172	Glycine max	no
SRR926173	Glycine max	no
SRR926174	Glycine max	no
SRR926175	Glycine max	no
SRR926176	Glycine max	no
SRR926177	Glycine max	no
SRR926178	Glycine max	no
SRR926342	Glycine max	no
SRR926347	Glycine max	no
SRR926348	Glycine max	no
SRR926391	Glycine max	no
SRR926392	Glycine max	no
SRR926393	Glycine max	no
SRR926394	Glycine max	no
SRR926395	Glycine max	no
SRR926396	Glycine max	no
SRR926398	Glycine max	no
SRR949966	Glycine max	no
SRR949967	Glycine max	no
SRR949968	Glycine max	no
SRR949969	Glycine max	no

SRA Run accession	Species	Is qualified?
SRR949970	Glycine max	no
SRR949971	Glycine max	no
SRR949972	Glycine max	no
SRR949973	Glycine max	no
SRR954983	Glycine max	no
SRR955300	Glycine max	no
SRR955301	Glycine max	no
SRR955302	Glycine max	no
SRR955406	Glycine max	yes
SRR976388	Glycine max	no
SRR976389	Glycine max	no
SRR976390	Glycine max	no
SRR976392	Glycine max	no
SRR976393	Glycine max	no
SRR976395	Glycine max	no
SRR976396	Glycine max	no
SRR346617	Solanum lycopersicum	yes
SRR346618	Solanum lycopersicum	yes
SRR346619	Solanum lycopersicum	yes
SRR346620	Solanum lycopersicum	yes
SRR346621	Solanum lycopersicum	yes
SRR346622	Solanum lycopersicum	yes
SRR346623	Solanum lycopersicum	no
SRR346624	Solanum lycopersicum	yes
SRR346625	Solanum lycopersicum	yes
SRR346626	Solanum lycopersicum	yes
SRR346627	Solanum lycopersicum	yes
SRR346628	Solanum lycopersicum	yes
SRR346629	Solanum lycopersicum	yes
SRR346630	Solanum lycopersicum	yes
SRR346631	Solanum lycopersicum	yes
SRR346632	Solanum lycopersicum	yes
SRR346633	Solanum lycopersicum	yes
SRR346634	Solanum lycopersicum	yes
SRR346635	Solanum lycopersicum	yes
SRR346636	Solanum lycopersicum	no
SRR389806	Solanum lycopersicum	no
SRR389807	Solanum lycopersicum	yes
SRR389808	Solanum lycopersicum	yes
SRR390315	Solanum lycopersicum	yes
SRR390328	Solanum lycopersicum	no
SRR390329	Solanum lycopersicum	yes
SRR390330	Solanum lycopersicum	no
SRR390331	Solanum lycopersicum	yes
SRR390335	Solanum lycopersicum	no

SRA Run accession	Species	Is qualified?
SRR390336	Solanum lycopersicum	yes
SRR404309	Solanum lycopersicum	no
SRR404310	Solanum lycopersicum	no
SRR404311	Solanum lycopersicum	no
SRR404312	Solanum lycopersicum	yes
SRR404313	Solanum lycopersicum	no
SRR404314	Solanum lycopersicum	yes
SRR404315	Solanum lycopersicum	no
SRR404316	Solanum lycopersicum	no
SRR404317	Solanum lycopersicum	no
SRR404318	Solanum lycopersicum	no
SRR404319	Solanum lycopersicum	no
SRR404320	Solanum lycopersicum	no
SRR404321	Solanum lycopersicum	no
SRR404322	Solanum lycopersicum	no
SRR404324	Solanum lycopersicum	yes
SRR404325	Solanum lycopersicum	no
SRR404326	Solanum lycopersicum	no
SRR404327	Solanum lycopersicum	no
SRR404328	Solanum lycopersicum	no
SRR404329	Solanum lycopersicum	no
SRR507782	Solanum lycopersicum	yes
SRR531827	Solanum lycopersicum	yes
SRR533977	Solanum lycopersicum	yes
SRR533979	Solanum lycopersicum	yes
SRR533980	Solanum lycopersicum	yes
SRR533981	Solanum lycopersicum	yes
SRR533982	Solanum lycopersicum	yes
SRR533987	Solanum lycopersicum	yes
SRR533988	Solanum lycopersicum	yes
SRR533997	Solanum lycopersicum	yes
SRR533998	Solanum lycopersicum	yes
SRR533999	Solanum lycopersicum	yes
SRR534000	Solanum lycopersicum	yes
SRR534001	Solanum lycopersicum	no
SRR534002	Solanum lycopersicum	yes
SRR546102	Solanum lycopersicum	yes
SRR546103	Solanum lycopersicum	no
SRR546104	Solanum lycopersicum	no
SRR546105	Solanum lycopersicum	no
SRR546106	Solanum lycopersicum	no
SRR546107	Solanum lycopersicum	no
SRR546108	Solanum lycopersicum	no
SRR546109	Solanum lycopersicum	no
SRR546110	Solanum lycopersicum	no

SRA Run accession	Species	Is qualified?
SRR546111	Solanum lycopersicum	yes
SRR546112	Solanum lycopersicum	no
SRR546113	Solanum lycopersicum	no
SRR546114	Solanum lycopersicum	no
SRR546115	Solanum lycopersicum	no
SRR546116	Solanum lycopersicum	no
SRR546117	Solanum lycopersicum	no
SRR567659	Solanum lycopersicum	yes
SRR567660	Solanum lycopersicum	yes
SRR567661	Solanum lycopersicum	yes
SRR567662	Solanum lycopersicum	yes
SRR567663	Solanum lycopersicum	yes
SRR567664	Solanum lycopersicum	yes
SRR567665	Solanum lycopersicum	yes
SRR567666	Solanum lycopersicum	yes
SRR567667	Solanum lycopersicum	yes
SRR567668	Solanum lycopersicum	yes
SRR567669	Solanum lycopersicum	yes
SRR567670	Solanum lycopersicum	yes
SRR567671	Solanum lycopersicum	yes
SRR567672	Solanum lycopersicum	yes
SRR567673	Solanum lycopersicum	yes
SRR567674	Solanum lycopersicum	yes
SRR567675	Solanum lycopersicum	yes
SRR567676	Solanum lycopersicum	yes
SRR567677	Solanum lycopersicum	yes
SRR567678	Solanum lycopersicum	yes
SRR567679	Solanum lycopersicum	yes
SRR567680	Solanum lycopersicum	yes
SRR567681	Solanum lycopersicum	yes
SRR567682	Solanum lycopersicum	yes
SRR567683	Solanum lycopersicum	no
SRR567684	Solanum lycopersicum	yes
SRR567685	Solanum lycopersicum	yes
SRR567686	Solanum lycopersicum	yes
SRR567687	Solanum lycopersicum	yes
SRR567688	Solanum lycopersicum	yes
SRR567689	Solanum lycopersicum	yes
SRR567690	Solanum lycopersicum	yes
SRR567691	Solanum lycopersicum	yes
SRR567692	Solanum lycopersicum	yes
SRR567693	Solanum lycopersicum	yes
SRR567694	Solanum lycopersicum	yes
SRR567997	Solanum lycopersicum	no
SRR567998	Solanum lycopersicum	yes

SRA Run accession	Species	Is qualified?
SRR567999	Solanum lycopersicum	yes
SRR568000	Solanum lycopersicum	yes
SRR570032	Solanum lycopersicum	yes
SRR570033	Solanum lycopersicum	yes
SRR570034	Solanum lycopersicum	yes
SRR570035	Solanum lycopersicum	yes
SRR570036	Solanum lycopersicum	yes
SRR570037	Solanum lycopersicum	yes
SRR570038	Solanum lycopersicum	yes
SRR570039	Solanum lycopersicum	yes
SRR570040	Solanum lycopersicum	yes
SRR570041	Solanum lycopersicum	yes
SRR570042	Solanum lycopersicum	yes
SRR570043	Solanum lycopersicum	yes
SRR570044	Solanum lycopersicum	yes
SRR570045	Solanum lycopersicum	yes
SRR570046	Solanum lycopersicum	yes
SRR570047	Solanum lycopersicum	yes
SRR570048	Solanum lycopersicum	yes
SRR570049	Solanum lycopersicum	yes
SRR570050	Solanum lycopersicum	yes
SRR570051	Solanum lycopersicum	yes
SRR570052	Solanum lycopersicum	yes
SRR570053	Solanum lycopersicum	yes
SRR570054	Solanum lycopersicum	yes
SRR570055	Solanum lycopersicum	yes
SRR570056	Solanum lycopersicum	yes
SRR570057	Solanum lycopersicum	yes
SRR570058	Solanum lycopersicum	yes
SRR570059	Solanum lycopersicum	yes
SRR570060	Solanum lycopersicum	yes
SRR570061	Solanum lycopersicum	yes
SRR768814	Solanum lycopersicum	no
SRR768815	Solanum lycopersicum	no
SRR768816	Solanum lycopersicum	no
SRR768817	Solanum lycopersicum	no
SRR768818	Solanum lycopersicum	yes
SRR768819	Solanum lycopersicum	no
SRR768822	Solanum lycopersicum	no
SRR768823	Solanum lycopersicum	no
SRR768824	Solanum lycopersicum	yes
SRR768825	Solanum lycopersicum	no
SRR768826	Solanum lycopersicum	no
SRR768827	Solanum lycopersicum	no
SRR768828	Solanum lycopersicum	yes

SRA Run accession	Species	Is qualified?
SRR768829	Solanum lycopersicum	yes
SRR768831	Solanum lycopersicum	no
SRR768832	Solanum lycopersicum	yes
SRR768833	Solanum lycopersicum	no
SRR768834	Solanum lycopersicum	no
SRR768835	Solanum lycopersicum	no
SRR768836	Solanum lycopersicum	no
SRR768837	Solanum lycopersicum	yes
SRR768838	Solanum lycopersicum	no
SRR768839	Solanum lycopersicum	no
SRR768840	Solanum lycopersicum	yes
SRR768841	Solanum lycopersicum	no
SRR768842	Solanum lycopersicum	no
SRR768843	Solanum lycopersicum	no
SRR768844	Solanum lycopersicum	no
SRR768845	Solanum lycopersicum	no
SRR768846	Solanum lycopersicum	no
SRR768847	Solanum lycopersicum	no
SRR768848	Solanum lycopersicum	no
SRR768849	Solanum lycopersicum	yes
SRR768850	Solanum lycopersicum	no
SRR768851	Solanum lycopersicum	no
SRR768852	Solanum lycopersicum	no
SRR768853	Solanum lycopersicum	no
SRR768854	Solanum lycopersicum	no
SRR768855	Solanum lycopersicum	no
SRR768856	Solanum lycopersicum	no
SRR768857	Solanum lycopersicum	no
SRR768858	Solanum lycopersicum	no
SRR768859	Solanum lycopersicum	no
SRR768860	Solanum lycopersicum	no
SRR768861	Solanum lycopersicum	no
SRR768862	Solanum lycopersicum	no
SRR768863	Solanum lycopersicum	yes
SRR768864	Solanum lycopersicum	no

Supplemental HTML. List of mRNA-Seq datasets downloaded and employed in current version of PODC (as of November, 2014). The SRA run accession numbers and plant species were listed. The third column shows whether each of them was qualified and actually-used or not.
